# Evaluation of noise excitation as a method for detection of hypernasality

**DOI:** 10.1016/j.apacoust.2022.108639

**Published:** 2022-03-15

**Authors:** Kat Young, Triona Sweeney, Rebecca R. Vos, Felicity Mehendale, Helena Daffern

**Affiliations:** a AudioLab, Department of Electronic Engineering, University of York, UK; b Speech at Home, Dublin, Ireland; c Speech and Audio Processing, Department of Electrical and Electronic Engineering, Imperial College London, UK; d Global Cleft Lip and Palate Research Programme, Global Health Research Centre, Usher Institute, University of Edinburgh, UK

**Keywords:** Hypernasality, Transfer function, Resonance

## Abstract

Hypernasality is a disorder where excess nasal resonance is perceived during speech, often as a result of abnormal coupling between the oral and nasal tracts known as velopharyngeal insufficiency (VPI). The most common cause of VPI is a cleft palate, which affects around 1 in 1650 babies, around ⅓ of whom have persistent speech problems after surgery. Current equipment-based assessment methods are invasive and require expert knowledge, and perceptual assessment methods are limited by the availability of expert listeners and differing interpretations of assessment scales. Spectral analysis of hypernasality within the academic community has resulted in potentially useful spectral indicators, but these are highly variable, vowel specific, and not commonly used within clinical practice.

Previous works by others have developed noise excitation technologies for the measurement of oral tract transfer functions using resonance measurement devices (RMD). These techniques provide an opportunity to investigate the structural system abnormalities which lead to hypernasality, without the need for invasive measurement equipment. Thus, the work presented in this study adapts these techniques for the detection of hypernasality. These adaptations include augmentation of the hardware and development of the software, so as to be suitable for transfer function measurement at the nostrils rather than the mouth (nRMD). The new method was tested with a single participant trained in hypernasal production, producing ‘normal’ and hypernasal vowels, and the recordings validated through a listening test by an expert listener and calculation of nasalance values using a nasality microphone. These validation stages indicated the reliability of the captured data, and analysis of the nRMD measurements indicated the presence of a systematic difference in the frequency range 2 to 2.5 kHz between normal and hypernasal speech. Further investigation is warranted to determine the generalisability of these findings across speakers, and to investigate the origins of differences manifesting in the transfer functions between conditions. This will provide new insights into the effects of nasal tract coupling on voice acoustics, which could in turn lead to the development of useful new tools to support clinicians in their work with hypernasality.

## 1 Introduction

Hypernasality is a type of speech disorder, defined as the occurrence of excess nasal resonance perceived during speech production. It can result from an abnormal coupling of the oral and nasal resonating cavities, due to incomplete closure of the velopharyngeal mechanism (soft palate and pharyngeal walls) [1] known as velopharyngeal insufficiency and/or incompetency (VPI). The most common cause of VPI is a cleft palate, which affects around 1 in 1650 babies, about ⅓ of whom have persistent speech problems after surgery [2]. In speech unaffected by VPI, the soft palate moves up and back in a ‘knee-like’ action to close firmly against the back wall of the pharynx, and the pharyngeal walls move to close against the velum [3]. Velopharyngeal insufficiency is indicative of a lack of tissue, while velopharyngeal incompetency typically results from a neurological disorder [4]. When VPI occurs, sound and air pass through the nasal tract, making speech more nasalised and difficult to understand. Depending on the type of VPI, treatment may include: surgery, in the case of an anatomical/structural defect (velopharyngeal insufficiency); or a combination of the surgery and speech therapy, especially where poor closure is due to a neurological control problem (velopharyngeal insufficiency) [3].

**Fig. 1:**
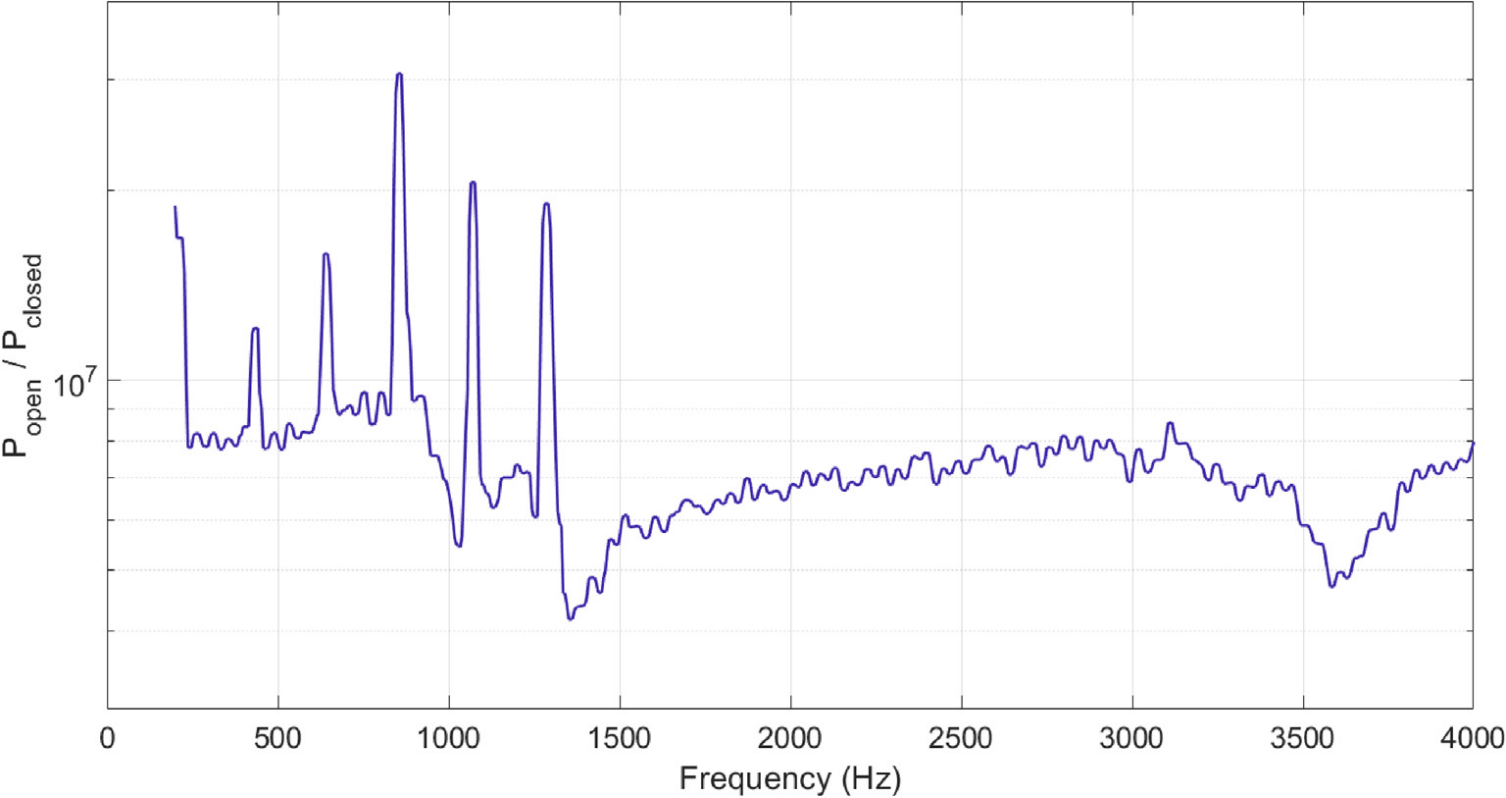
An example measurement using the RMD at the mouth for a held /ɑ/. The voice source harmonics are visible as sharp peaks superimposed on the transfer function of the tract. Note the use of a logarithmic y-axis, as used by Vos et al. [41].

Velopharyngeal function can be assessed through numerous techniques, summarised by Bettens et al. [5] into groupings of direct and indirect methods. Direct methods are often invasive and can expose the patient to radiation, but allow visualization of the closing mechanism through nasoendoscopy, videofluoroscopy, dynamic magnetic resonance imaging (MRI), lateral cephalometric radiographic analysis, computed tomography (CT), and ultrasound. Indirect methods infer velopharyngeal activity using aerodynamic or acoustic measurements. Aerodynamic measurements, such as the flow of air or pressure (measured using the Aerophonoscope system [6] and Perci system [7], respectively) allow the function of the closure mechanism to be inferred, however the equipment being used can still be invasive or uncomfortable. Techniques that involve the placement of accelerometers on the surface of the nose and throat have been developed, measuring the ratio between their signal amplitudes in the case of the Horii Oral Nasal Coupling (HONC) Index [8], and the duration of the signals in the case of the Nasality Oral Ratio Meter (NORAM) [9]. The commercially-available Nasometer (consisting of a pair of microphones on either side of an acoustic separator) is the most commonly-used clinical measurement tool. It measures the oral nasal acoustic ratio [10] (also referred to as the nasalance value) which describes the ratio of nasal energy to nasal-plus-oral energy, expressed as a percentage.

Whilst these acoustic measures are sometimes utilised by clinicians, they require expert knowledge to be used correctly, and function only as a supplementary guide to the primary assessment criteria. In clinical settings, perceptual analysis by an experienced speech and language therapist (SLT) is still deemed to be the gold standard in assessment of hypernasality (and other speech disorders), especially considering the resource needed for treatments: if the speech problem is not perceivable, there is less of a need to introduce interventions [11], [12]. However, relying solely on perceptual analysis can be limited by a number of factors, including availability of SLTs, inter-rater and intra-rater reliability, use of different ratings scales and interpretations of those scales, and varying correlation with non-perceptual measurements [11], [13].

Whilst mostly academic rather than utilised within clinical practice, spectral analysis of acoustic measurements has revealed numerous valuable approaches [14], [15], [16], [17], [18], [19], [20], [21], [22], [23], [24]. Much of the work involving spectral feature analysis incorporates machine learning within the workflow, however, rather than feeding the results of feature-analysis into prediction algorithms, Mathad et al. [25] have trained a Deep Neural Network (DNN) classifier to learn the patterns of hypernasal and non-hypernasal speech in order to separate the two.

Spectral analysis to assess hypernasality is, however, influenced by the voice source of the speaker and, unless direct measurement occurs in parallel, the association between spectral feature and a structural velopharyngeal behaviour can only be inferred. In addition, spectral changes will often be speaker and vowel specific. Transfer functions provide an ideal means with which to investigate articulatory and acoustic phenomena as they are not influenced by the source [26]. A number of well-documented spectral characteristics associated with coupling between the nasal and oral tracts have been identified through analysis of transfer functions [17], [26], [27], [28], [29], [30], [31], [32], [33].

Alongside an increase in amplitude in the lower frequencies (approximately 250 Hz) [17], [26], [27], [28], [29], perturbations to the first formant (F1) above this frequency region have consistently been observed across hypernasal speakers and vowels [28]. These perturbations include increasing in bandwidth [17], [28], [30], [33], a reduction in amplitude [27], [28], [30], and, due to the introduction of a dip, F1 splitting into two peaks [27], [30], [33]^1^. Other spectral perturbations have been observed above F1, which are less consistent across speaker and vowel, including additional dip/peak pairs throughout the spectrum [26], [27], [29], [30], a flattening of the spectrum where the amplitudes of the peaks become more similar [27], [30], [31], a decrease in the amplitude of the second formant (F2) [21], [30], and an increase in amplitude in the frequency region 2–4 kHz relative to F1 [31], [33]. The majority of previous work considering spectral features through transfer functions has relied on computational models, simulations, or the excitation of 3D-printed vocal tracts measured from MRI images. These methods don’t capture the potential impact of other features that might affect the transfer function in live measurement such as the mucous membrane, the absorption properties of the tract walls, and the paranasal cavities, which have been shown in some research to alter the transfer function [31], [32], [33].

If obtainable from live subjects in a non-invasive way, transfer functions could be practically helpful to SLTs, providing a meaningful description of the structural system without the need for invasive and expensive measurement techniques such as MRI. There is a body of work conducted on the measurement of vocal tract transfer functions of live subjects through excitation at the glottis using stimuli such as swept tones, gaussian noise and pseudo-random pulse series [37], [38], however, these methods require articulatory positions to be held static for long periods of time and are uncomfortable [39]. Epps et al. [39] developed a method which allows for measurement of the vocal tract resonances at the mouth during phonation. This method presents the underlying vocal tract resonances and the acoustic output of the voice as distinct and separate, and has been successfully implemented for the detection and training of resonance tuning (where the vocal tract is adjusted to more closely match the resonances with the harmonics in the voice [40]) in singers [36], [40], [41], [42], [43], [44] and for analysis of vowel pronunciation [45].

The aim of this study was to adapt these techniques for analysis of vocal tract resonances in live subjects for the detection of hypernasality, working with the Resonance Measurement Device (RMD) developed from the works described above. The RMD has almost exclusively been implemented with sustained vowels, whilst hypernasality is typically assessed through perceptual ratings of connected speech. If this method can successfully provide indicators of hypernasality, it could be developed further into a useful tool from which to objectively consider structural behaviours within specific vowels for specific speakers alongside perceptual assessment of connected speech.

This paper describes the development of a novel method employing the RMD for acoustic measurement at the nose (nRMD) to assess hypernasality, with a case study of a single participant producing normal^2^ and nasalised vowels. Section 2 describes the methods used for the experiment, including participant, tasks, recording equipment, and data processing. Section 3 presents the validation processes conducted to ensure accuracy and consistency of production of nasality by the participant. Section 4 presents and discusses the results of the nRMD analysis comparing normal and nasalised vowels. Limitations of these results are discussed in Section 5, with conclusions summarised in Section 6.

## 2 Methods

Ethical approval for the study (ref: Young080320) was obtained from the Physical Sciences Ethics Committee (PSEC) at the University of York (UK).

### 2.1 Participant

A participant without a history of VPI, and therefore capable of producing both normal and hypernasal speech, was needed for comparison between hypernasal and normal speech of the same speaker. A case study was conducted with a single participant (co-author K.Y.). The participant was 28, was assigned female at birth and had typical oestrogen-based development, and has a Northern English accent. They had no history of nasal regurgitation when eating or drinking and had no history of speech difficulties suggestive of VPI. On intra-oral examination, their palate showed no evidence of a cleft, the uvula was not bifid, velar elevation was symmetrical and there was no evidence of anterior levators.

It was not deemed appropriate to use patients for this study due to the ethical implications and the complexities of cleft speech in patients, whereby the presentation usually includes more than one of many abnormal parameters, including nasality, nasal airflow and articulation. When evaluating a novel method of hypernasality detection, as in the case of this paper, it was essential that we studied imitated speech samples that had only hypernasality as distinct from clinical samples with multiple other confounding variables and with variable levels of severity of hypernasality. Patients can also show variation in the levels of hypernasality depending on speed of speech and on degree of tiredness. Such clinically challenging variations, while part of clinical practice, would not provide optimum samples for this early testing work.

As part of the validation methods, an expert listener (co-author T.S., a Consultant Cleft Specialist Speech and Language Therapist with 40 years experience in management of speech disorders related to Cleft Palate) trained the participant and conducted the perceptual assessment (Section 3.1).

### 2.2 Participant training

A series of participant training sessions were conducted with the expert listener over the course of several weeks, along with regular individual practice sessions, to ensure that the participant could consistently produce nasalised vowels and words akin to that produced by a patient with hypernasality. Iterative assessments were made by the expert listener to aid the training process, and a blind listening test by the expert listener (T.S.) indicated the successful repeat production of hypernasal vowels and words based on the binary classifier of ‘normal’ and ‘hypernasal’ speech.

### 2.3 Tasks

After the initial period of training and assessment, 13 CVC words were selected by the expert listener for use in the main experiment (listed in Table 1). The English spelling of a word and the hypernasal version were provided (for example, ‘mee’ is the hypernasal pronunciation of ‘bee’). These words were selected to give wide coverage of English vowels (without creating unduly long data collection or assessment processes), and were easy to teach and learn to produce with hypernasality.

**Table 1:** The 13 words chosen for inclusion in the main experiment, the vowel in IPA notation for participant’s accent, and the hypernasal version. * denotes the presence of an additional ‘t’ in the recording. As both the perceptual rating and nasalance value (discussed in Section 3) were consistent with hypernasal production, the data was retained.

Normal	Vowel in IPA notation for participant’s accent (Northern English)	Hypernasal
Bee	/i/	Mee
Den	/ɛ/	Nen
Bell	/ɛ/	Mell
Ban	/a/	Man
Barn	/ɑ/	Marn
Dawn	/ɔ/	Mawn
Doll	/ɒ/	Noll
Bow	/ə/	Mow
Boot	/u/	Moon(t)*
Dune	/u/	Nune
Bun	/ʌ/	Mun
Burn	/ɜ/	Nurn
Book	/ʊ/	Noong

The participant repeated each CVC word at a normal volume and consistent pitch in both normal and hypernasal speech three times using two sets of measurement equipment (detailed further in Section 2.4). While pitch was not tightly controlled, the participant did not stray far from their normal speaking pitch (fundamental frequency of approximately 250 Hz). The participant did not have any symptoms of an upper respiratory infection at the time of data recording. The vowel portion of each CVC word was extended to approximately two seconds in accordance with the requirements of the RMD. For each word, there were two speech conditions, two measurement setups and three takes, giving a total of 12 measurements per word.

As both the acoustic and the resonance measurements required the placement of instrumentation in close proximity to the upper lip and nose, it is not possible to measure using both setups simultaneously. Therefore, the participant alternated between the two (described in Table 2). Using the equipment in such a way reduced the likelihood of an unintended systematic difference between the acoustic measurements and the resonance measurements. It also reduced the fatigue of the participant, not requiring sustained production of accurate hypernasal speech for long periods of time.

**Table 2:** The pattern of recording used to include all combinations of the two speech conditions, two measurement conditions and three takes for each word.

Recording #	Take #	Acoustic measurement	Resonance measurement
1	1	Normal	
2	1	Hypernasal	
3	1		Normal
4	1		Hypernasal
5	2	Normal	
6	2	Hypernasal	
7	2		Normal
8	2		Hypernasal
9	3	Normal	
10	3	Hypernasal	
11	3		Normal
12	3		Hypernasal

### 2.4 Recording setup

Two measurement conditions were required to allow for comparison between established clinical methods and the new measurement device (the RMD). All measurements were recorded in a quiet room at the participant’s house (noise floor of approximately 40 dBSPL (A-weighted)).

#### 2.4.1 Acoustic measurement setup

Acoustic data were captured for stimuli for the listening test and to calculate nasalance. Two sets of microphones were used simultaneously: a nasality microphone where the acoustic signals are captured using two microphones separated by a plate which is pressed against the face, and a distant reference microphone (audio-technica AT2020 [46] frequency response available at [47]) placed 1 m from the participant, at the height of the participant’s mouth (1.55 m). Nasalance, calculated using the software accompanying the nasality microphone, is the ratio of the energy in the nasal signal to the total energy (nasal + oral) [10]. Pilot testing confirmed that the presence of a nasality microphone did not interfere with the audio captured at the reference microphone. The audio was recorded using a Zoom F8 multitrack field recorder [48] at 44.1 kHz, 16 bit and stored as mono PCM .wav files. The gain levels were set to give a good recording level whilst speaking at a normal volume.

The method requires the device to be placed at the opening to the nostril, which results in the occlusion of the majority of one nostril by the microphone. Whilst this doesn’t affect the results of the current study as this condition was consistent across comparisons, it might affect interpretation of the transfer functions as representing the nasal tract acoustics.

The nasality microphone (from Rose Medical [49]) was calibrated and positioned according to the manufacturer instructions, with the acoustic separator plate pressed firmly to the face between the upper lip and nose. The audio was recorded using the associated USB adapter and software (Nasalance Viewer) on a MSI GP64 Leopard 8RE Windows 10 laptop. The default values of 8 kHz and 16 bit were used, and the audio stored as stereo PCM .wma files. Stereo files are created as each of the two microphones are recorded onto a channel (nasal on the left channel, oral on the right channel). During recording, a mirror was used to ensure the same positioning each time. If the position was noticed to have deviated substantially, both the normal and the hypernasal utterances were repeated.

#### 2.4.2 Resonance measurement device setup

The RMD, initially developed by Epps et al. [39] and used by others [36], [40], [41], [42], [43], [45] involves exciting the vocal tract at the mouth using a synthesised broadband noise signal, while simultaneously recording the response with a co-located microphone. The MATLAB control software used in this study was originally developed by Henrich et al. [40] and adapted by Vos et al. [41]. Briefly, the software calculates the unit-less ratio between the impedance of the tract and that of the radiation field (referred to as P_open_ and P_closed_, respectively). This is analogous to the transfer function with harmonics of the voice source superimposed on top as sharp peaks, an example of which is shown in Fig. 1.

Briefly, the RMD comprises a loudspeaker with an impedance-matching horn connected to a flexible hose, with a microphone mounted adjacent to the open end of the hose [41], [50]. The excitation signal is pseudorandom broadband noise, consisting of a number of synthesised equally spaced harmonics with the phases randomised. The spacing and number of these harmonics is derived from the required frequency range, the sampling frequency and the desired frequency resolution, which also define the time duration of the analysis window. Here, a frequency range of 200 Hz to 4 kHz, a sampling frequency of 44.1 kHz and a 2^12^-point FFT were used, giving a total of 355 harmonics spaced 10.77 Hz apart and an analysis window of 93 ms (2^12^ samples at 44.1 kHz).

A number of adjustments were made to the equipment described by Vos et al. [41], [50] for the purposes of this study; changing the location of the measurement, and changing the calibration process.

**Fig. 2:**
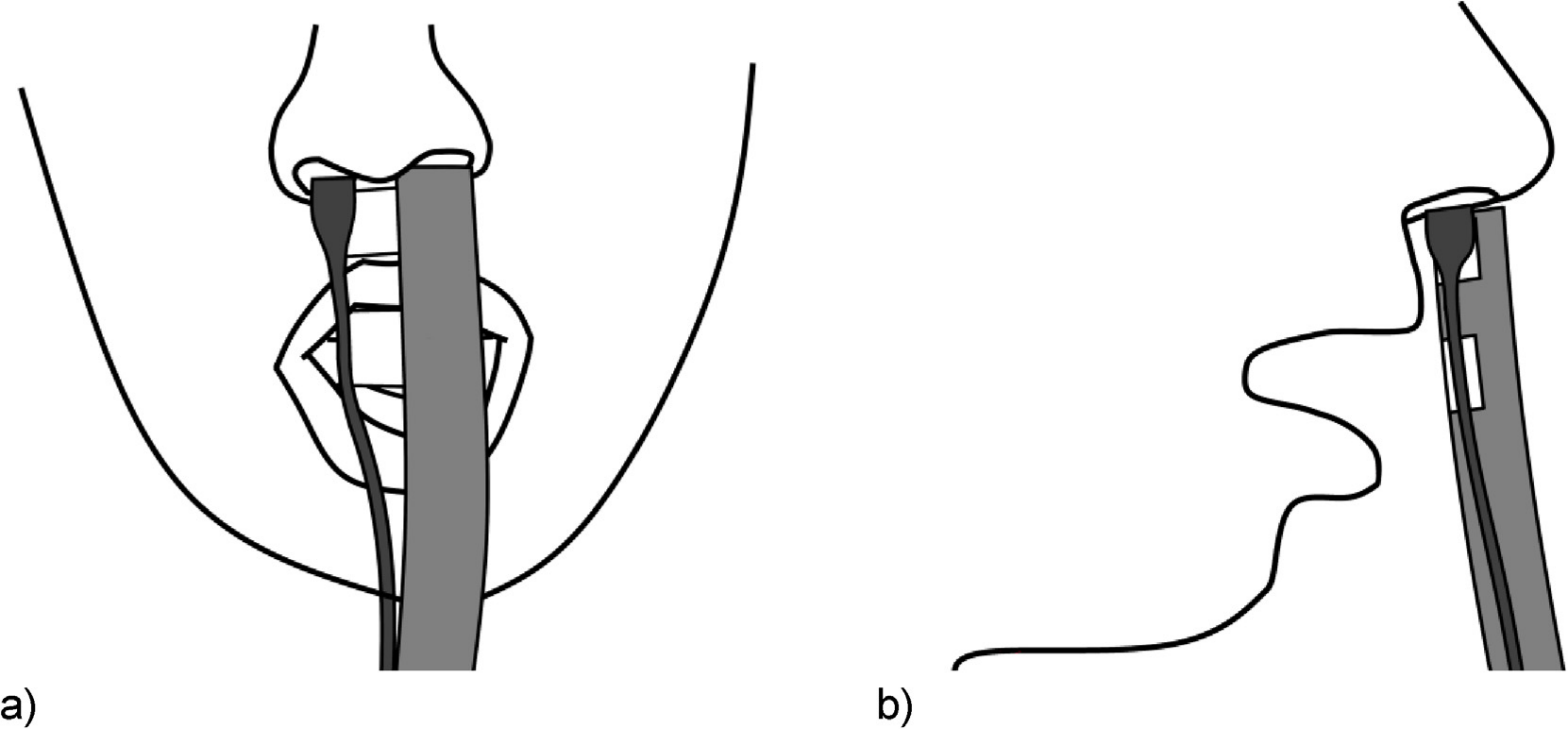
Front (a) and side (b) view of the RMD using the configuration development for this study: at the nostrils rather than the mouth (nRMD).

Positioning the RMD at the mouth produces limited data for CVC words (as facial and articulatory movement cause blurring of the data), and it does not perform particularly well with closed vowels [50]. For this application, the RMD was adapted to allow for measurement at the nostrils (nRMD) (shown in Fig. 2). A pair of spacing blocks were added between the hose and microphone to increase their inter-centre-point distance to 15 mm, and the participant rested the uppermost spacer on their nasal septum during measurement. The tube and microphone are placed externally to the nostril, although depending on the shape of the individuals nose this may include very slight occlusion to part of the individuals nostril, and therefore might be considered slightly invasive. However neither the tube or microphone are intended to be inserted into the nostril outlet, only to rest on the septum.

The previous version also used a calibrated excitation signal to calculate the ratio between the impedance of the vocal tract and that of the radiation field. This calibration stage involved playing the excitation signal with the RMD positioned against their closed lips, and the software adjusted the harmonics to create a signal with a flat frequency response [41]. Practically, this accounts for the spectral effects that are not of interest, for example those caused by the contours of the face or the system response of the equipment. This calibration stage was not implemented in this study for two reasons. Firstly, it is rather more complicated when calibrating for measurement at the nostrils than at the mouth, as it is not possible to ‘close’ the tract in the same way as closing the mouth, and raises the question of what articulator positions should be used to achieve the most ‘neutral’ position. Second, the primary interest here is the difference between two transfer functions of the same speaker rather than absolute transfer function, so the effect of any additional spectral information is not of interest. Therefore, the excitation signal used here consisted of synthesised harmonics with randomised phases, but without the calibration stage as described by Vos et al. [41]. The resulting measurement will be referred to simply as ‘the ratio’, rather than P_open_ over P_closed_ as in previous work.

For measurement, the RMD was positioned within reach of the participant, and connected to the same MSI GP64 Leopard 8RE Windows 10 laptop as the nasality microphone using an RME Fireface UCX interface [51] with a Nobsound NS 01G Pro amplifier (discontinued, replaced by [52]). The output level was adjusted such that the level at the end of the hose was 90 dBSPL (A-weighted). The participant positioned the microphone and hose at their nostrils (as described previously), triggered the playback of the five second excitation signal using MATLAB, and spoke during the playback. The MATLAB software captures the result at the microphone and stores the data as a .mat file in double precision. As with the acoustic recordings, a mirror was used to ensure the same positioning each time. If the position was noticed to have deviated substantially, both the normal and the hypernasal utterances were repeated.

### 2.5 Data processing

Previous versions of the associated RMD software [40], [41], [43], [44] were adapted in order to store the measurement prior to processing. One second excerpts with the most consistent pitch and amplitude were selected using MATLAB R2020a [53].

The transfer function of the nasal/vocal tract system was obtained from these excerpts using the methods described by others [39], [41], [44] using software first developed by Henrich et al. [40]. The time domain signal was split into a number of identical-length rectangular analysis windows, the FTT taken of each window, and the average spectrum across all windows was calculated. The ratio was then calculated between this average spectrum and that of the excitation signal.

As discussed previously, the length of the analysis windows is related to the sampling frequency and the FFT point value used. Here, 2^12^ samples at 44.1 kHz gives a window duration of 93 ms. With the one second excerpts, this gives 11 windows across which the average is calculated.

A recent real-time implementation of the software [44], [54] included an algorithm to remove the voice content harmonics which appear as sharp peaks in the signal. To avoid greater numerical differences between transfer functions as a result of slightly mismatched voice harmonic content, this algorithm was adapted and incorporated into the offline processing workflow used in this study. Briefly: the spectrum is smoothed and peaks with a particular prominence value (the height above and position relative to other peaks [55]) and distance apart are detected (prominence of 1 standard deviation, frequency distance of 65 Hz); peaks with a width less than a set threshold (100 Hz) are removed and the data replaced with a straight line; the spectrum is smoothed again.

**Fig. 3:**
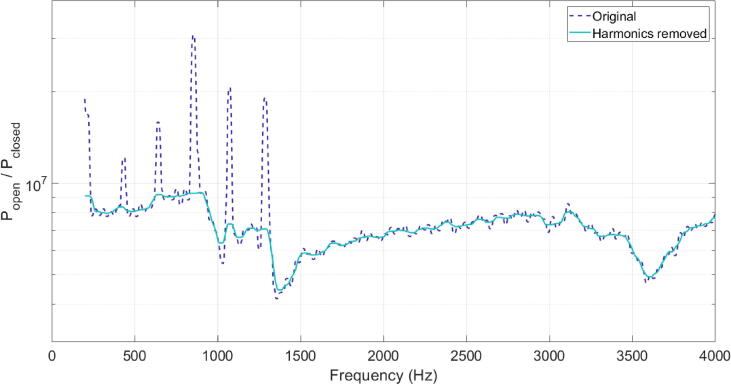
An example of the result of the harmonic content removal algorithm. The example data is plotted as in (/ɑ/, dotted line) with the result of the harmonic removal algorithm also plotted (solid line). The narrow peaks have been substantially reduced in amplitude.

The result of processing using this algorithm is shown in Fig. 3, where the amplitude of narrow peaks corresponding to voice source harmonics have been substantially reduced in the data plotted in Fig. 1.

## 3 Validation

A number of validation stages were included in the protocol to ensure that the participant was able to produce consistent normal and hypernasal speech which aligned with clinical assessment. These stages consisted of a perceptual listening test, comparison of nasalance values across takes, and analysis of the consistency of the nRMD measurements. The results of the listening test were also compared to the nasalance values, to ensure that utterances were assessed consistently. As it was not possible to directly compare the data captured using the two sets of measurement equipment, these validation stages, in combination with the recording order discussed in Section 2.4, ensured that consistency was maintained across all 12 recordings for each word.

### 3.1 Perceptual listening test

The expert listener completed a perceptual listening test to validate the ability of the participant in producing and repeating hypernasal speech. Both whole CVC words and isolated vowels taken from the CVC words were included, in order to include the scenario that would be encountered in a clinical setting (whole CVC words) as well as the stimuli needed for the nRMD analysis (isolated vowels).

#### 3.1.1 Listening test method

The reference microphone recordings were used as stimuli for the listening test. Two sets of listening test stimuli were extracted using Praat v5.3.04 [56]: whole words, and one-second portions of each vowel chosen for consistency in pitch and amplitude. These words and vowel tokens were then processed using MATLAB: normalised to avoid clipping, windowed with a 10-sample half-Hann window at the onset and offset, formatted into loops to repeat the same token three times with one second of silence between, and saved as 44.1 kHz, 16 bit, mono PCM .wav files. Sound files of looped vowel tokens were approximately eight seconds long, while files of looped word tokens were approximately 15 s long.

The audio files were presented over SennheiserHD 205 headphones using Qualtrics (running on Safari on a Macbook Air, running macOS Catalina). The expert listener could replay files as required with no limit on the total amount of time taken, and to go back to a previous presentation and change their answer if they wished. Files were presented in two groups: first looped isolated vowel tokens, then the whole words, to avoid influence caused by hearing the isolated vowel in the context of the full word. There were 78 tokens in each group (presented in a random order within the group), with 26 (33%) randomly selected to be repeated for intra-listener reliability testing. This gave a total of 208 presentations, which the expert listener assessed over a duration of approximately 90 min.

Hypernasality is usually rated using a multi-point scale such as the Temple Street Scale described by Sweeney and Sell [11] but a binary classifier was needed for the purpose of testing the system. Therefore, a three-choice forced-choice answer paradigm was used within the listening test, where the listener chose between “Hypernasality is present”, “Hypernasality is absent”, or “Could not tell” for each token. For the isolated vowels, the corresponding word was listed, as the intention was to assess the nasality of the vowel, not identify the vowel itself.

The listening test results were compared to the intention of the participant (that is, whether the utterance was intended to be hypernasal or not) for each audio token and the percentage that matched was calculated. Consistency of the expert listener was also assessed using percentage agreement and Cohen’s Kappa across repeated tokens, calculated using MATLAB.

#### 3.1.2 Listening test results

Over all tokens presented, there was 95.7 % agreement (where the intended mode of production matched the rating given) between the participant and the expert listener (199/208 tokens), with agreement of 100 % and 91.3 % when split into whole words and isolated vowels, respectively. The results for whole words are perhaps unsurprising, as these included the nasalised consonants and were therefore considerably easier to detect as hypernasal. Splitting the isolated vowels by speech condition, there was agreement of 92.0 % and 90.7 % for hypernasal and normal vowels, respectively. The nine tokens rated either as “Could not tell” or with a mismatch between participant intention and expert listener assessment were: three ‘barn’, two ‘book’, two ‘burn’, and one each of ‘bun’ and ‘dune’.

Looking at the consistency of the expert listener across takes of the same audio tokens, there was an agreement of 94 % and a Cohen’s Kappa value of 0.88. There was no pattern between the tokens rated differently across the takes.

These results indicate both that the participant was able to produce normal and hypernasal speech as desired, and the expert listener was consistent in their assessment.

### 3.2 Participant consistency: Nasalance

Nasalance measures were used in addition to perceptual assessment to ensure that the participant was able to produce normal and hypernasal speech within the usual nasalance range for each condition, and that the productions were consistent within each group.

#### 3.2.1 Nasalance analysis

**Fig. 4:**
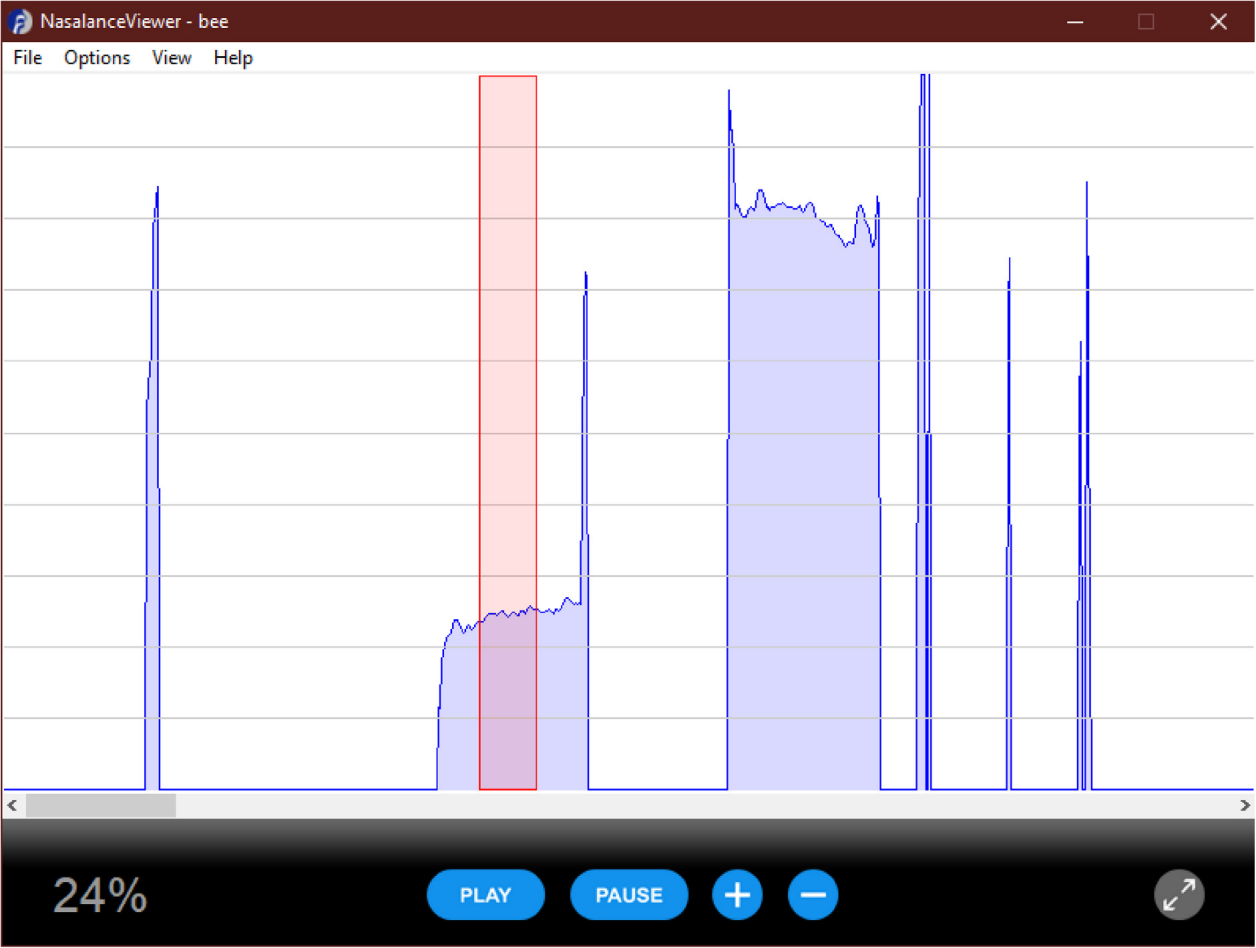
Screenshot of the Nasalance Viewer software demonstrating the calculation of nasalance within a marker region. The region corresponding to the portion used in the first take of ‘bee’ for normal speech is shown in red; the average nasalance within the region is shown in the bottom left corner.

The nasalance of each vowel was calculated using the associated software Nasalance Viewer [49], with the band-pass filter enabled (centre frequency of 500 Hz, bandwidth of 350–650 Hz). The vertical cursors were positioned to match the excerpts used in the listening test, and the mean nasalance (referred to from this point as ‘nasalance’) calculated for the marked area. This is shown in Fig. 4, for the example of ‘bee’. The mean and standard deviation of the nasalance values for each of the three repeats of each vowel were calculated, and compared to values in the literature [57], [58].

#### 3.2.2 Nasalance results

**Fig. 5:**
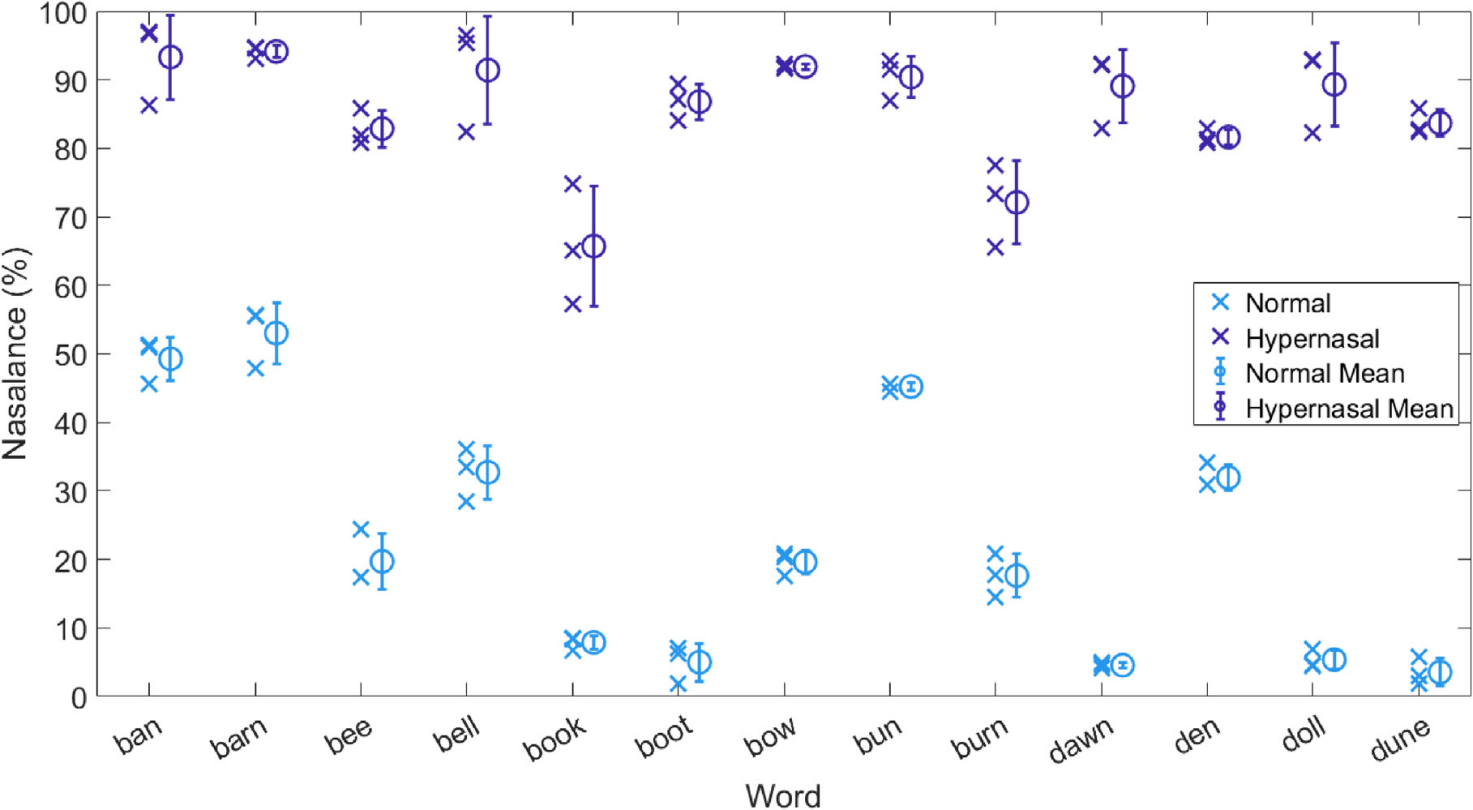
The nasalance value for each token (x) and the mean (o) and standard deviation (whiskers) of each set of three takes. The normal and hypernasal speech conditions are plotted in different colours to reveal two distinct groups.

The nasalance value for each token is plotted as a cross (x) in Fig. 5. The mean and standard deviation for each set of three repeats is marked using circles and whiskers. The colour denotes the intention of the participant, where blue is normal speech and purple is hypernasal speech.

As expected, the nasalance values differ between vowels, and two distinct groupings can be observed, with consistently greater values of nasalance for the tokens which the participant intended to be hypernasal. A two-sample Kolmogorov-Smirnov test indicates a significant difference between the two distributions (p < 0.05). However, the nasalance values for ‘ban’, ‘barn’, and ‘bun’ are greater than would be expected for normal speech (for example, Lewis et al. [58] found mean values of 10–20 % for vowels spoken by participants without velopharyngeal dysfunction). This may be due to anticipatory articulation of the /n/ sound. Therefore, these three words are excluded from the subsequent analysis. Exclusion of these words increases the percentage match between participant and expert listener discussion in 3.1.2, 3.3, 4, 5 to 96.9 % (154/159 tokens).

The standard deviation values for normal speech are generally less than the 5–7 % found for sentence stimuli by Sweeney et al. [57] and the 7–10 % found for vowels by Lewis et al. [58]. However, the standard deviation values here may be artificially low due to the small number of participants. The standard deviation values for hypernasal speech are comparable or slightly larger than those for normal speech; this is to be expected as the participant was not familiar with the production of the speech condition prior to beginning the training for this experiment. The small standard deviations in nasalance values indicate that the participant was consistent within repeats of the same word and speech condition.

### 3.3 Assessment consistency

**Fig. 6:**
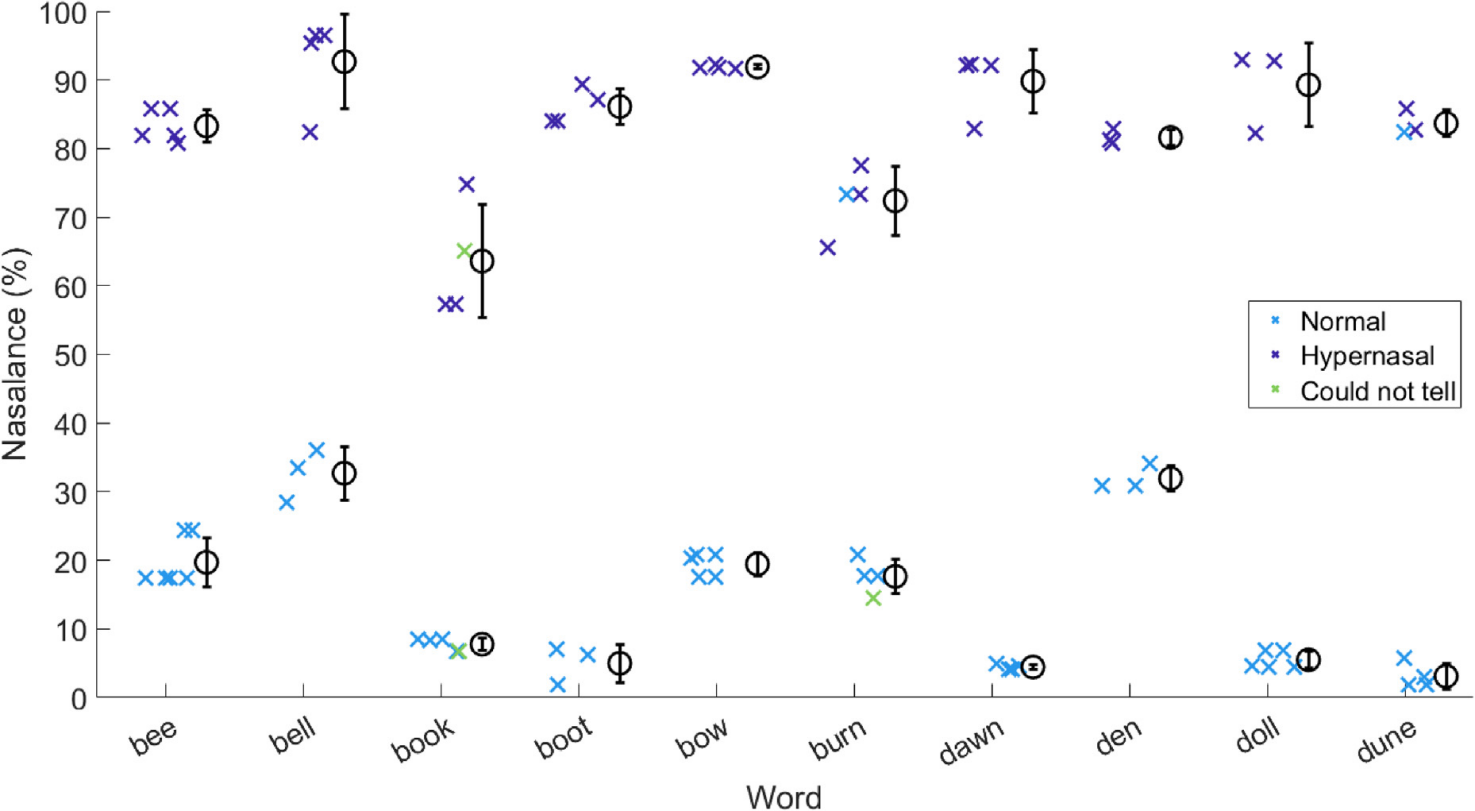
The nasalance value for each token of each vowel (x), the mean of each set of three (o) and the standard deviation of each set of three (whiskers). Colour represents the rating given by the expert listener during the perceptual listening test.

The nasalance values and perceptual ratings for each token were compared to determine the level of consistency between assessment methods. Fig. 6 shows the nasalance values as plotted in Fig. 5 with additional markers for repeated tokens, but here colour represents the rating given by the expert listener during the listening test. The markers have been given a small random spacing on the x-axis to allow the takes to be seen more clearly, and as takess are included, the mean and standard deviation values are slightly different to those plotted above.

On the whole, there are two distinct groups either side of approximately 50% nasalance. Fitting a one-predictor logistic regression to the data using MATLAB indicated that nasalance is a significant predictor of being rated as hypernasal (p < 0.01), as described in Eq. (1), and gives the halfway point as 49.33 %.

(1) Predictedlogitof(RATINGHYPERNASAL)=-4.2588+0.0863×(NASALANCE)

Therefore, the greater the nasalance value, the more likely to be rated as hypernasal, which indicates agreement between the intentions of the participant, the nasalance value as calculated using the software, and the perceptual ratings given by the expert listener.

### 3.4 Participant consistency: resonance

The consistency between takes of the same vowel within speech condition as measured using the nRMD setup was analysed to ensure that these measurements were also comparable across takes.

#### 3.4.1 Resonance consistency analysis

The correlation was calculated using MATLAB between transfer functions corresponding to takes of the same vowel within speech condition. Three pairs of transfer functions exist for each speech condition (as order does not matter, **_3_C_2_** = 3), therefore giving six correlation coefficients per vowel. Spearman’s rank correlation was used as the data was not normally distributed. The transfer functions such as that plotted with a solid line in Fig. 3 (with harmonic content removed) were used to avoid influencing the results by including highly-correlated harmonic voice content.

#### 3.4.2 Resonance consistency results

Table 3 lists the correlation coefficients between the pairs of transfer functions within each speech condition for each vowel (where HN and N denotes hypernasality and normal speech, respectively, and the number denotes the take), and the mean value for each speech condition. All p values are<0.01. Generally, the coefficients are greater for normal speech than for hypernasal speech, mirroring the consistency of nasalance values discussed in Section 3.2.2. The minimum and maximum values are 0.80 and 0.98, respectively, giving a range of 0.18. These results indicate strong similarity between the measurements within each speech condition for each vowel.

**Table 3:** Correlation coefficients between pairs of transfer functions within speech condition groups (where HN and N denotes hypernasality and normal speech, respectively, and the number denotes the take) for each vowel, and the mean value within each speech condition group. * denotes p < 0.01.

Vowel	Correlation coefficients between transfer functions						Mean within	
	HN1 – HN2	HN1 – HN3	HN2 – HN3	N1 – N2	N1 – N3	N2 – N3	HN pairs	Normal pairs
Bee	0.91*	0.91*	0.90*	0.94*	0.97*	0.98*	0.91	0.97
Bell	0.82*	0.81*	0.84*	0.93*	0.95*	0.97*	0.83	0.95
Book	0.92*	0.88*	0.85*	0.96*	0.96*	0.98*	0.88	0.97
Boot	0.89*	0.89*	0.91*	0.94*	0.98*	0.94*	0.90	0.95
Bow	0.92*	0.88*	0.89*	0.92*	0.96*	0.92*	0.90	0.93
Burn	0.95*	0.86*	0.89*	0.89*	0.91*	0.95*	0.90	0.92
Dawn	0.80*	0.90*	0.91*	0.94*	0.92*	0.97*	0.87	0.94
Den	0.90*	0.85*	0.92*	0.98*	0.94*	0.95*	0.89	0.96
Doll	0.95*	0.91*	0.92*	0.92*	0.96*	0.95*	0.93	0.94
Dune	0.93*	0.84*	0.90*	0.95*	0.96*	0.97*	0.89	0.96

## 4 Results and discussion

The results of the validation methods described in Section 3 indicate that the participant is capable of consistently producing both normal and hypernasal vowels and words, and that the hypernasal speech would be assessed as such by currently-used clinical methods. Therefore, the main research question within this study can be addressed: whether any features of hypernasality are indicated in the results captured by the nRMD.

### 4.1 Correlation across speech conditions

The correlation across speech conditions was calculated using MATLAB between pairs of transfer functions for takes of the same vowel across the two conditions. In contrast to the correlation coefficients calculated in Section 3.3, comparing transfer functions across speech conditions should give smaller correlation coefficients than within condition analysis. As before, Spearman’s rank correlation and the transfer functions with the voice harmonics removed were used.

As there are nine combinations for each vowel (**_6_C_2_** = 15, excluding the six described in Section 3.3), it is impractical to list all results here. As an example, Table 4 shows the nine values for the vowel in ‘dawn’. All p values are below 0.01, and the mean value is 0.71 (as subsequently listed in Table 5). All correlation coefficients are below those calculated for within speech groups for this vowel, and a two-sample Kolmogorov-Smirnov test indicates a significant difference between the two distributions (p < 0.05). Table 6 (in Supplementary Data) lists the coefficients for the remaining words.

**Table 4:** Correlation coefficients between pairs of transfer functions across speech condition groups for ‘dawn’ (where HN and N denotes hypernasality and normal speech, respectively, and the number denotes the take). * denotes p < 0.01.

Transfer function pair	N1 - HN 1	N1 - HN 2	N1 - HN 3	N2 - HN 1	N2 - HN 2	N2 - HN 3	N3 - HN 1	N3 - HN 2	N3 - HN 3
Correlation	0.67*	0.61*	0.65*	0.78*	0.71*	0.72*	0.80*	0.72*	0.74*

**Table 5:** Mean correlation coefficients between pairs of transfer functions within each speech condition group (left and centre columns, as in , where speech condition is denoted as HN and N for hypernasality and normal speech, respectively) and between pairs of transfer functions across speech condition groups (right-most column).

Word	Mean correlation within		Mean correlation across speech condition groups
	HN pairs	Normal pairs	
Bee	0.91	0.97	0.90
Bell	0.83	0.95	0.74
Book	0.88	0.97	0.70
Boot	0.90	0.95	0.84
Bow	0.90	0.93	0.57
Burn	0.90	0.92	0.81
Dawn	0.87	0.94	0.71
Den	0.89	0.96	0.75
Doll	0.93	0.94	0.79
Dune	0.89	0.96	0.74

Table 5 lists the mean correlation value across speech condition for each vowel, alongside the mean values within speech condition as previously listed in Table 3. For each vowel, the correlation across conditions is smaller than the correlation within conditions.

**Fig. 7:**
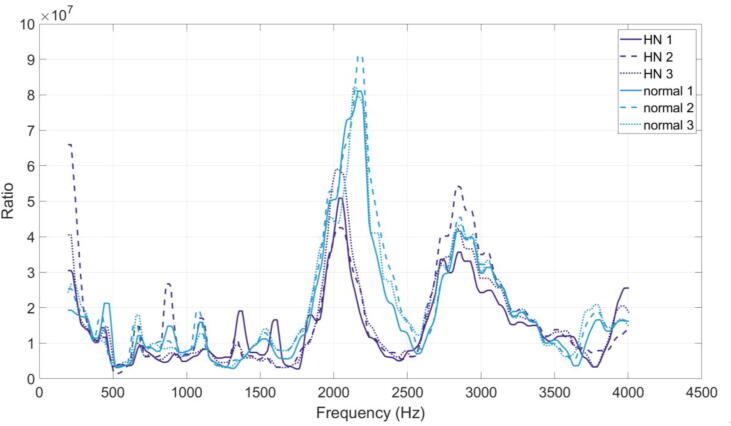
The six transfer functions measured using the nRMD at the nostrils for the vowel in ‘dawn’. Colour denotes speech condition, line style denotes repetition.

Visual inspection of the transfer functions gives some insight into these greater values. Fig. 7 shows the six transfer function measurements (with harmonic content removed) for the vowel in ‘dawn’, where a linear y-axis has been used (rather than the logarithmic y-axis used previously) to allow differences to be more clearly seen. The peaks in the transfer functions at approximately 2 kHz and 3 kHz are likely the resonances of the nasal tract, as discussed by Havel et al. [59], although not present in these transfer functions is the resonance found at approximately 700 Hz. This may be due in part to coupling of the nasal tract with the oral tract, as the velopharyngeal opening was sealed in [59]. There is a noticeable visible difference between normal and hypernasal measurements in the frequency region 1.8 kHz to 2.5 kHz, but little difference across the rest of the spectrum, which may be contributing to the greater correlation values both within and across speech conditions. A Wilcoxon signed-rank test (paired, two-sided) indicated that there was a statistically significant difference between the normal and hypernasal measurements for this vowel (Z = 11.58, p < 0.05).

### 4.2 Differences between speech conditions

The differences between speech condition measurements shown above for the vowel in ‘dawn’ are generally representative of the other vowels (included in Supplementary Data). Wilcoxon signed-rank tests (paired, two-sided) indicated a statistically significant difference between the normal and hypernasal measurements for each vowel (p < 0.05). Whilst the transfer function shape differs between vowels, there are generally differences between speech conditions in the frequency region 1.8 kHz to 2.5 kHz, with very little difference below and above this region. Predominantly, the differences manifest as a change in amplitude and frequency of a peak within this region, where the peak is at a greater centre frequency and has a greater amplitude in the normal speech condition. The amount of this difference varies with vowel, and does not appear for all vowels: for example, the peaks in the normal and hypernasal conditions are similar in amplitude and centre frequency for the six measurements for ‘bee’ (Fig. 10), and for ‘book’ (Fig. 12) and ‘burn’ (Fig. 15) the peaks are at different centre frequencies but are of similar amplitudes across speech conditions.

**Fig. 8:**
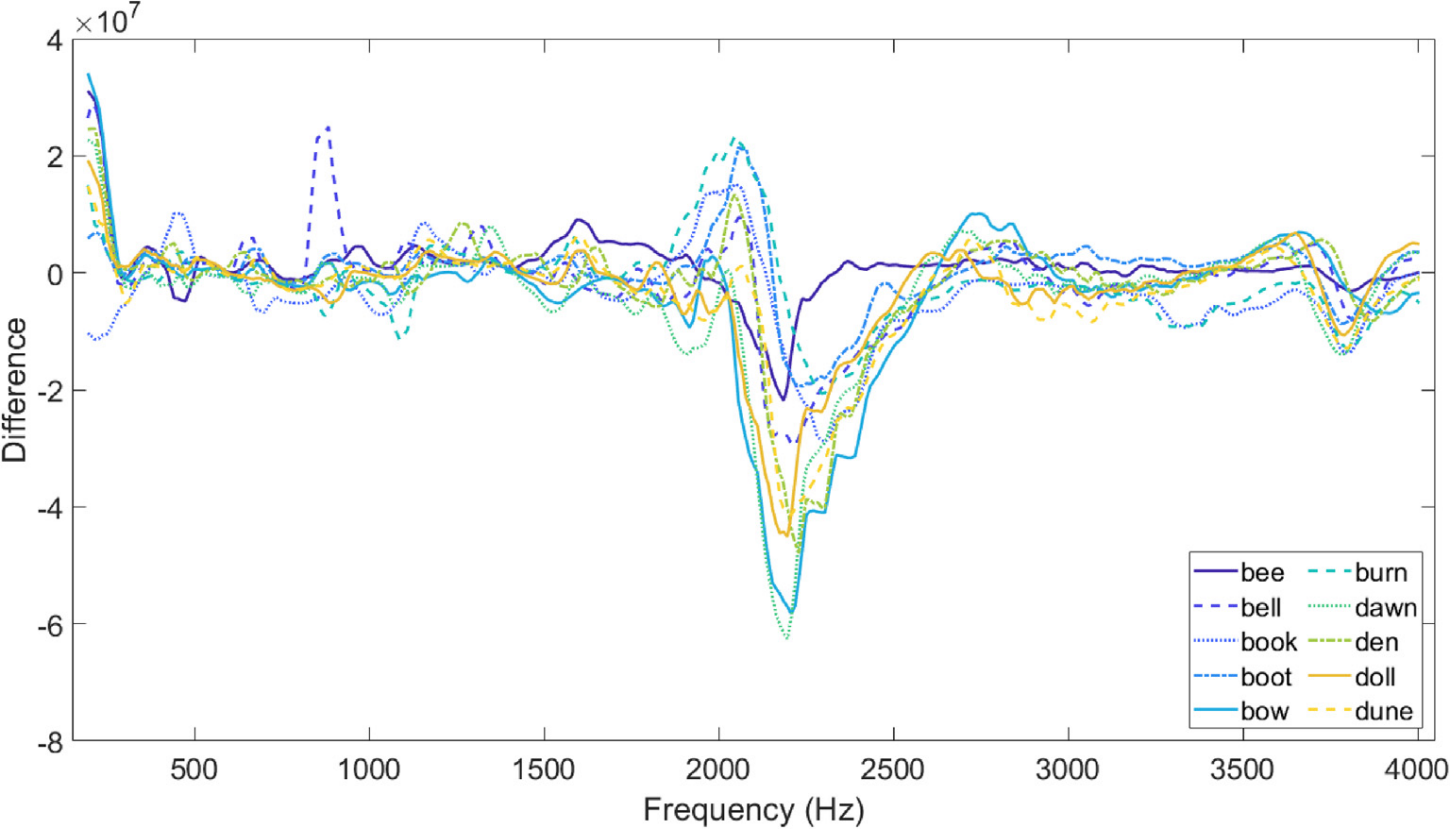
Difference between the average hypernasal and the average normal transfer function for each vowel, denoted using colour and linestyle for clarity.

Fig. 8 shows the difference across frequency between the average hypernasal transfer function and the average normal transfer function for each vowel (where vowel is denoted using both colour and linestyle for clarity). Here, a negative value indicates that the normal condition measurement has a greater value than the hypernasal condition at that frequency point. Where there is a difference in both centre frequency and amplitude of a peak, a large dip is present. A peak-dip feature (such as that for ‘bow’) is a result of the peak changing only in centre frequency.

**Fig. 9:**
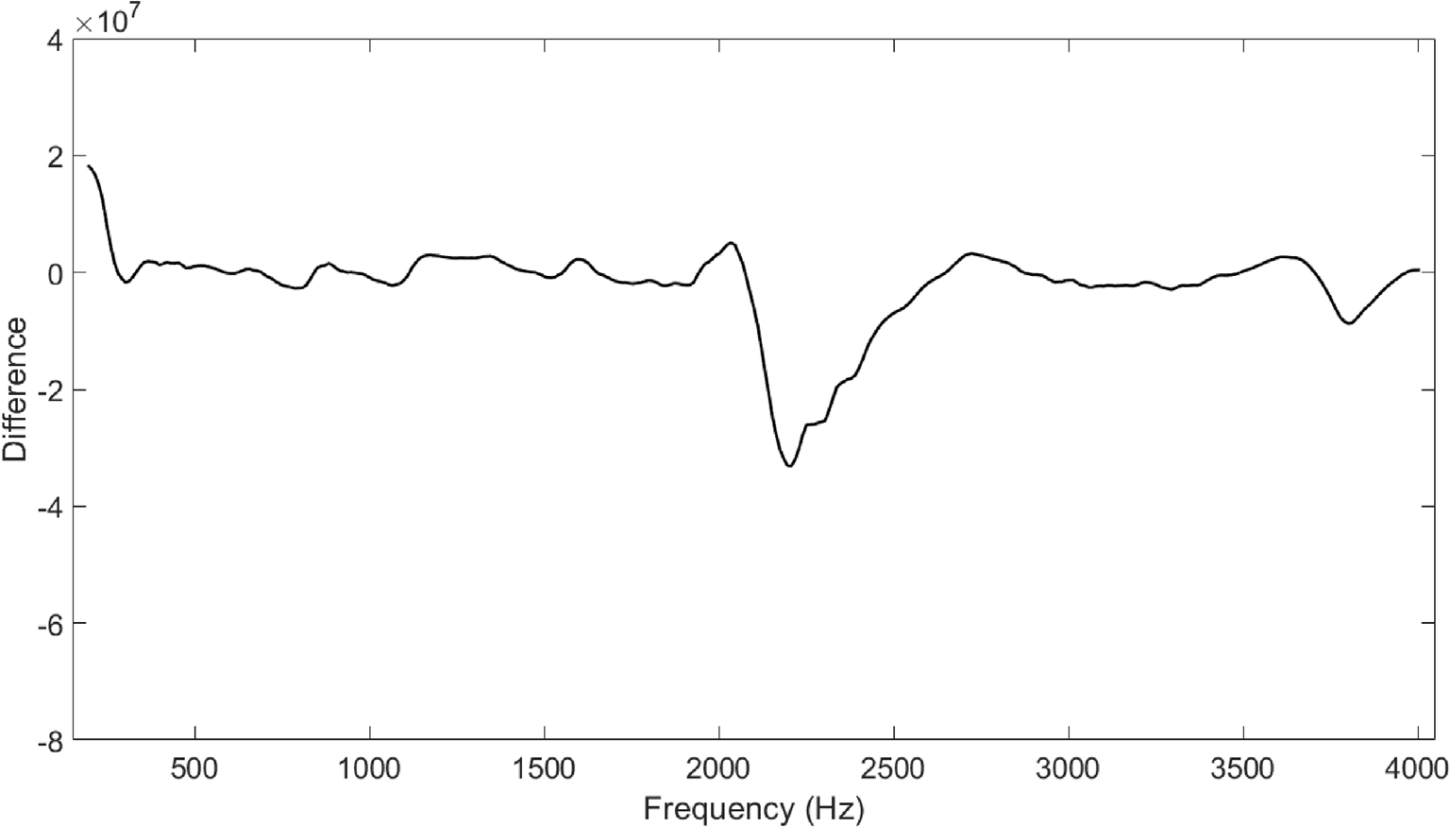
Difference between the average of all hypernasal measurements and the average of all normal measurements.

Fig. 9 shows the unit-less numerical difference across frequency between the average of all hypernasal measurements and the average of all normal measurements. Although the differences captured by the nRMD appear to vary with vowel, calculating this difference shows that there is a systematic difference between the normal and the hypernasal speech using this measurement technique. On average, hypernasal vowels have a peak of smaller amplitude and smaller centre frequency within the region 2 kHz to 2.5 kHz, and have more low frequency energy in the region of 200 Hz (as suggested in the literature [17], [26], [27], [28], [29]).

### 4.3 nRMD-based indications of hypernasality

The results presented in this study indicate the presence of a systematic difference between normal and hypernasal speech conditions that can be detected using the nRMD. There is evidence of a difference within the frequency region 2 kHz to 2.5 kHz, whereby the peak present in the normal speech condition decreases in centre frequency and amplitude for the hypernasal condition. Whether this is a generalisable ‘feature’ of hypernasality needs further investigation. If it is found to be relevant across speakers then the manifestation of this spectral characteristic also requires future study to understand the origin of this spectral feature; for example, rather than simulations of transfer functions of oral tract (which are usual in research, for example: [26], [27], [31]), simulations of the transfer function of the nasal tract with altered termination of the tube into the oral cavity.

Other spectral changes discussed in the literature, such as perturbations in the frequency regions around F1 and F2 [17], [26], [27], [28], [29], [30], [31], [33], are not clearly apparent in the results reported in this study. This is most likely due to the measurement technique and the resulting data: relating the changes in spectrum in the F1 and F2 regions is perhaps not relevant here, as the oral tract is not being excited in a way that would generate visible F1 and F2 characteristics. With the nRMD, which measures at the nostrils rather than the mouth, the ‘normal’ condition is the natural configuration of the nasal tract (with the natural amounts of nasal-oral coupling that occur in speech), and the ‘hypernasal’ condition increases the coupling between the nasal and oral tracts. As a result of this, the transfer functions acquired in this study are not oral tract transfer functions, which makes direct comparison to existing literature inappropriate.

## 5 Limitations

The results presented in this study are derived from a single participant, as the planned additional data collection with trained expert participants became untenable due to the restrictions related to the COVID-19 pandemic. In order to mitigate this limitation extra validation procedures were undertaken to ensure the reliability of the data. While this implies that the results are not generalisable at this stage, they do give indication of the likely success of the measurement system. Future work should investigate whether such differences are present across multiple speakers, and consider in more detail the implications of measuring the transfer function through the nose, as well as the impact of vowel variation on the resulting transfer function using this method. There has been much discussion in the literature as to the vowel-dependent nature of nasality-related spectral changes, and it would be of great interest to investigate this further using the nRMD.

Due to the data processing method and windowing applied in the analysis, the nRMD does not perform well with CVC words and connected speech and so the current study was performed on sustained vowels. Whilst the system in its current form might provide useful means with which to further understand the acoustic impact of oral, nasal tract coupling and even a promising supplementary tool for SLTs, in practice hypernasality is typically assessed using connected speech rather than sustained vowels. Further adaptations would make exploration of using nRMD on connected speech possible, however this was outside the scope of the current study.

## 6. Future work

It would be valuable to repeat the current methodology in a study which recruits a group of expert SLT participants who are able to reproduce normal and hypernasal speech. This would allow cross-speaker comparison of the features of the transfer functions obtained by the nRMD that indicate nasalance. Once the generalisability of these features across expert speakers has been established, a study involving speakers from both diagnosed hypernasal and normal populations is needed. This will establish whether hypernasality can be assessed from absolute features in the nRMD transfer functions across populations, or if features indicative of hypernasality only emerge as comparative data within subjects who are able to produce both speech conditions. More resource-heavy studies, with collection of 3D Magnetic Resonance Imagining of the vocal tract in parallel with nRMD transfer functions, would connect the transfer function features with articulatory gestures, from which simulation models could test the observed relationships. These additional steps would also support detailed analysis of the transfer functions themselves, to compare features obtained from the nRMD directly with other measures of nasality which are taken either at the mouth [37], [38] or from 3D models [31], [33].

Additional development of the control software is required to enable analysis of connected speech using the nRMD. A high frequency resolution was required in this work as the exact nature of any indicative features within the transfer function was unknown. Using a frequency resolution of 10.77 Hz gave an analysis window of 93 ms. As this is longer than the duration over which speech is assumed to be quasi-stationary (typically 20–30 ms, in some cases up to 80 ms [60]), taking the average over a number of windows overcomes variation in the transfer function within the analysis window, and is the cause of the nRMD being best suited for the measurement of sustained vowels. In order to analyse connected speech, the averaging stage must be removed and the analysis window duration must be shortened, and the resultant decrease in frequency resolution must be accepted.

If universal markers of nasality across subjects can be identified, especially through analysis of connected speech, there is potential for the nRMD to be developed further as a clinical tool that could support SLT and cleft palate surgeons as a non-invasive and easy to use hypernasality detector, which is also cheaper than current alternatives and may also support diagnosis of VPI in the future.

## 7. Conclusions

This study developed a new method for the detection of hypernasality through broadband noise excitation at the nostrils (nRMD), adapting an acoustic resonance measurement device (RMD) used in previous work. A pilot study with a single participant producing a number of CVC words in both normal and hypernasal speech conditions was conducted. To test data consistency and reliability, nasality ratings were obtained through perceptual evaluation and acoustic measures were made using a nasality microphone, in addition to the capture of the same tasks using the nRMD. These measures suggest that the data is robust in terms of the speaker successfully producing repetitions of normal and hypernasal speech.

Evidence of a systematic difference between normal and hypernasal speech was observed in the resulting transfer functions from the nRMD, with a clear peak between 2 kHz and 2.5 kHz changing in both centre frequency and amplitude between the two conditions. The results of the case study suggest that the developed methodology is successful in providing useful data representative of produced hypernasality by a speaker. While this study was limited to a single participant, the findings are encouraging and suggest that broadband excitation at the nostrils could be a valuable tool for the measurement of hypernasality. Future work is needed to explore the generalisability of nasalance features obtained using the nRMD technique, as well as developing the technique further to be effective on running speech. With these next steps, this technique has potential to be developed for use in clinical settings to support SLTs and cleft palate surgeons in their diagnosis and treatment of hypernasality.

### CRediT authorship contribution statement

**Kat Young:** Methodology, Software, Investigation, Writing – original draft, Writing – review & editing, Data curation, Project administration. **Triona Sweeney:** Conceptualization, Methodology, Writing – original draft, Supervision, Writing – review & editing, Funding acquisition, Project administration. **Rebecca R. Vos:** Conceptualization, Methodology, Resources, Writing – review & editing. **Felicity Mehendale:** Conceptualization, Validation, Writing – review & editing. **Helena Daffern:** Conceptualization, Writing – review & editing.

## Declaration of Competing Interest

The authors declare that they have no known competing financial interests or personal relationships that could have appeared to influence the work reported in this paper.

## Footnotes

1 Meanings and notations of the term ‘formants’ in the literature have not been standardised as in [34] and may refer to peaks in the output spectrum measured at the mouth [35] and/or the resonances of the vocal tract which give rise to formants [36] interchangeably.

2 The term ‘normal’ is used in this paper to refer to the usual speech production of the participant with natural levels of nasalisation.
