# Supplementary material for: Evaluation of noise excitation as a method for detection of hypernasality
Source: Appl Acoust. 2022 Mar 15;190:108639. doi: 10.1016/j.apacoust.2022.108639 (PMC8872831; doi:10.1016/j.apacoust.2022.108639)
Supplement: Supplementary data 1 [file mmc1.docx]

# Supplementary Data

| **Vowel** | **Correlation coefficients between transfer functions** | | | | | | | | |
| --- | --- | --- | --- | --- | --- | --- | --- | --- | --- |
|  | N1 - HN 1 | N1 - HN 2 | N1 - HN 3 | N2 - HN 1 | N2 - HN 2 | N2 - HN 3 | N3 - HN 1 | N3 - HN 2 | N3 - HN 3 |
| Bee | 0.88* | 0.87* | 0.90* | 0.86* | 0.90* | 0.89* | 0.91* | 0.92* | 0.93* |
| Bell | 0.74* | 0.77* | 0.75* | 0.70* | 0.68* | 0.67* | 0.80* | 0.74* | 0.78* |
| Book | 0.74* | 0.73* | 0.75* | 0.63* | 0.63* | 0.65* | 0.74* | 0.69* | 0.71* |
| Boot | 0.88* | 0.81* | 0.87* | 0.81* | 0.81* | 0.85* | 0.81* | 0.84* | 0.84* |
| Bow | 0.58* | 0.55* | 0.52* | 0.58* | 0.56* | 0.49* | 0.65* | 0.58* | 0.58* |
| Burn | 0.88* | 0.83* | 0.87* | 0.84* | 0.81* | 0.83* | 0.71* | 0.78* | 0.78* |
| Dawn | 0.67* | 0.61* | 0.65* | 0.78* | 0.71* | 0.72* | 0.80* | 0.72* | 0.74* |
| Den | 0.85* | 0.87* | 0.80* | 0.68* | 0.73* | 0.66* | 0.69* | 0.76* | 0.74* |
| Doll | 0.83* | 0.71* | 0.78* | 0.83* | 0.70* | 0.80* | 0.85* | 0.73* | 0.83* |
| Dune | 0.77* | 0.74* | 0.72* | 0.80* | 0.77* | 0.78* | 0.72* | 0.68* | 0.69* |
| Table 6: Correlation coefficients between pairs of transfer functions across speech condition groups (where HN and N denotes hypernasality and normal speech, respectively, and the number denotes the take) for each vowel. * denotes p<0.01. | | | | | | | | | |

| 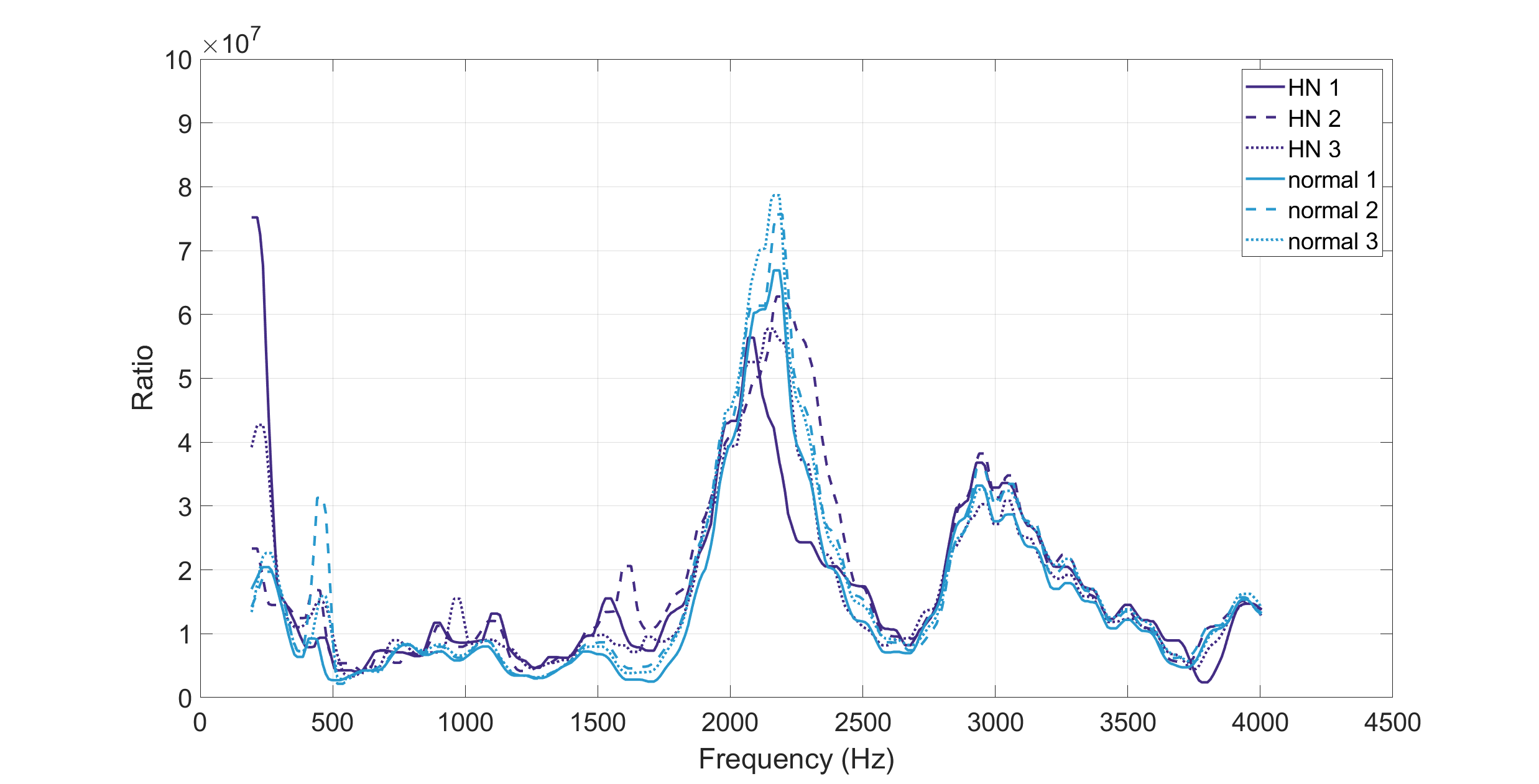 |
| --- |
| Figure 10: Transfer functions measured using the RMD at the nostrils for the vowel in ‘bee’. Colour denotes speech condition, line style denotes repetition. |

| 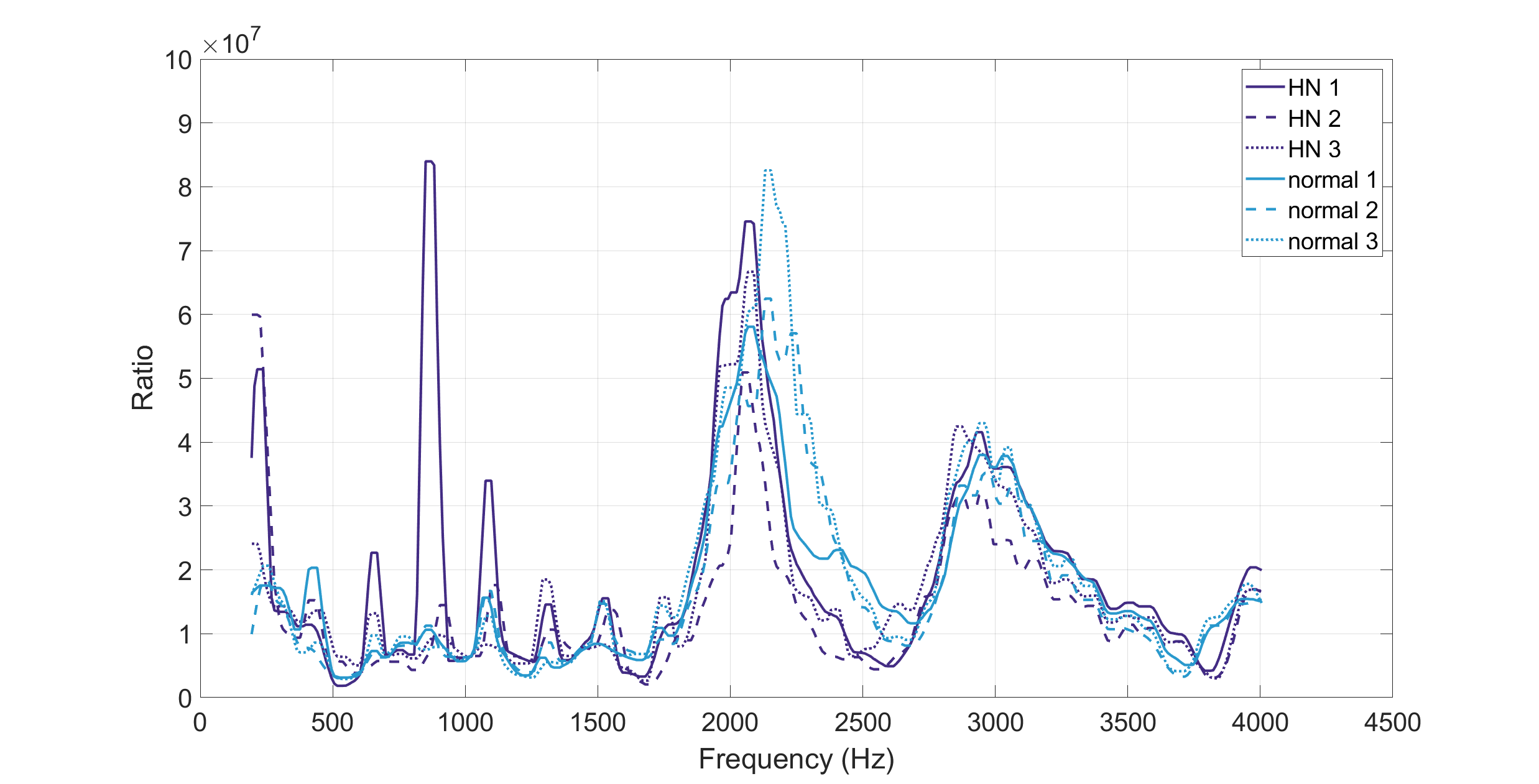 |
| --- |
| Figure 11: Transfer functions measured using the RMD at the nostrils for the vowel in ‘bell’. Colour denotes speech condition, line style denotes repetition. |

| 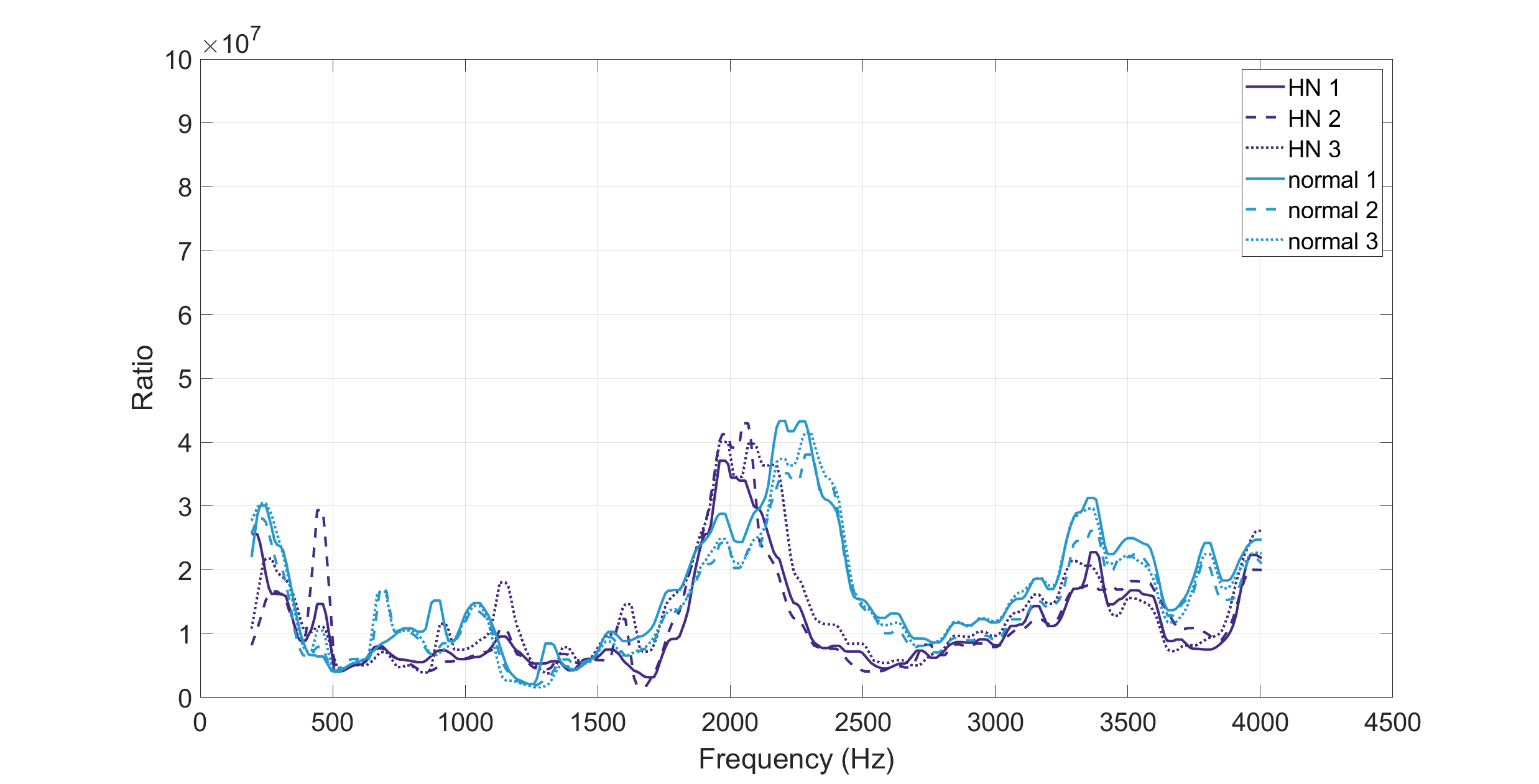 |
| --- |
| Figure 12: Transfer functions measured using the RMD at the nostrils for the vowel in ‘book’. Colour denotes speech condition, line style denotes repetition. |

| 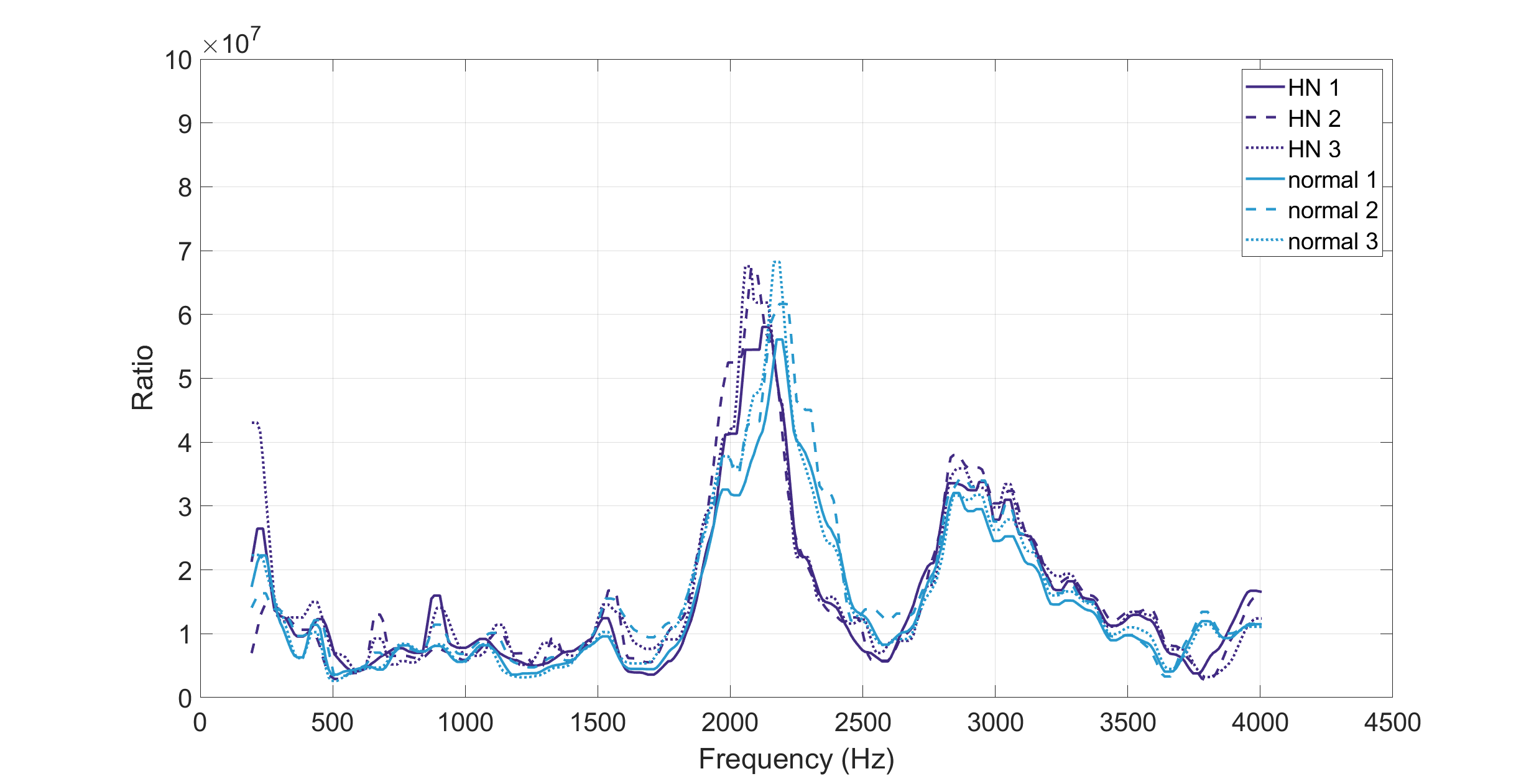 |
| --- |
| Figure 13: Transfer functions measured using the RMD at the nostrils for the vowel in ‘boot’. Colour denotes speech condition, line style denotes repetition. |

| 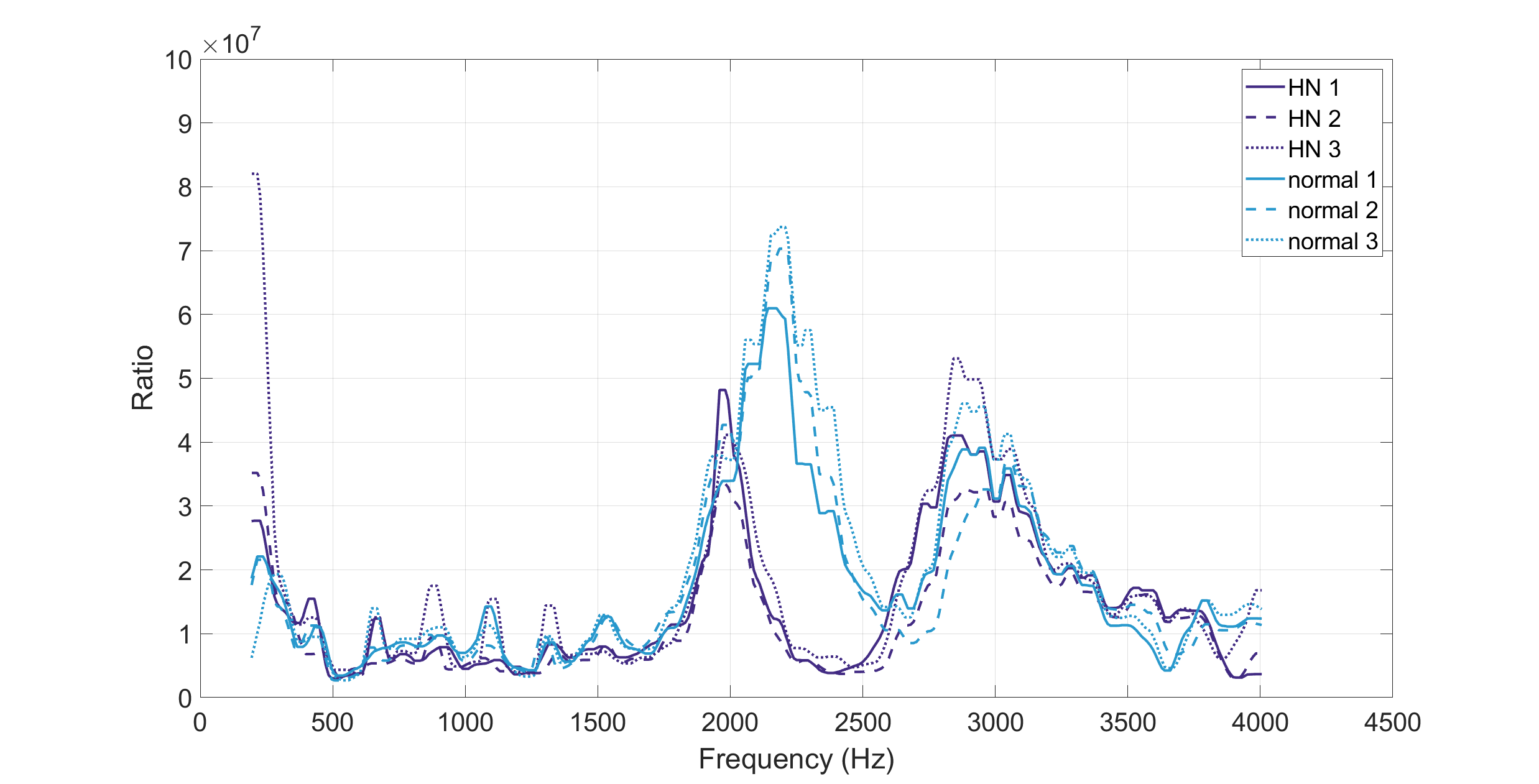 |
| --- |
| Figure 14: Transfer functions measured using the RMD at the nostrils for the vowel in ‘bow’. Colour denotes speech condition, line style denotes repetition. |

| 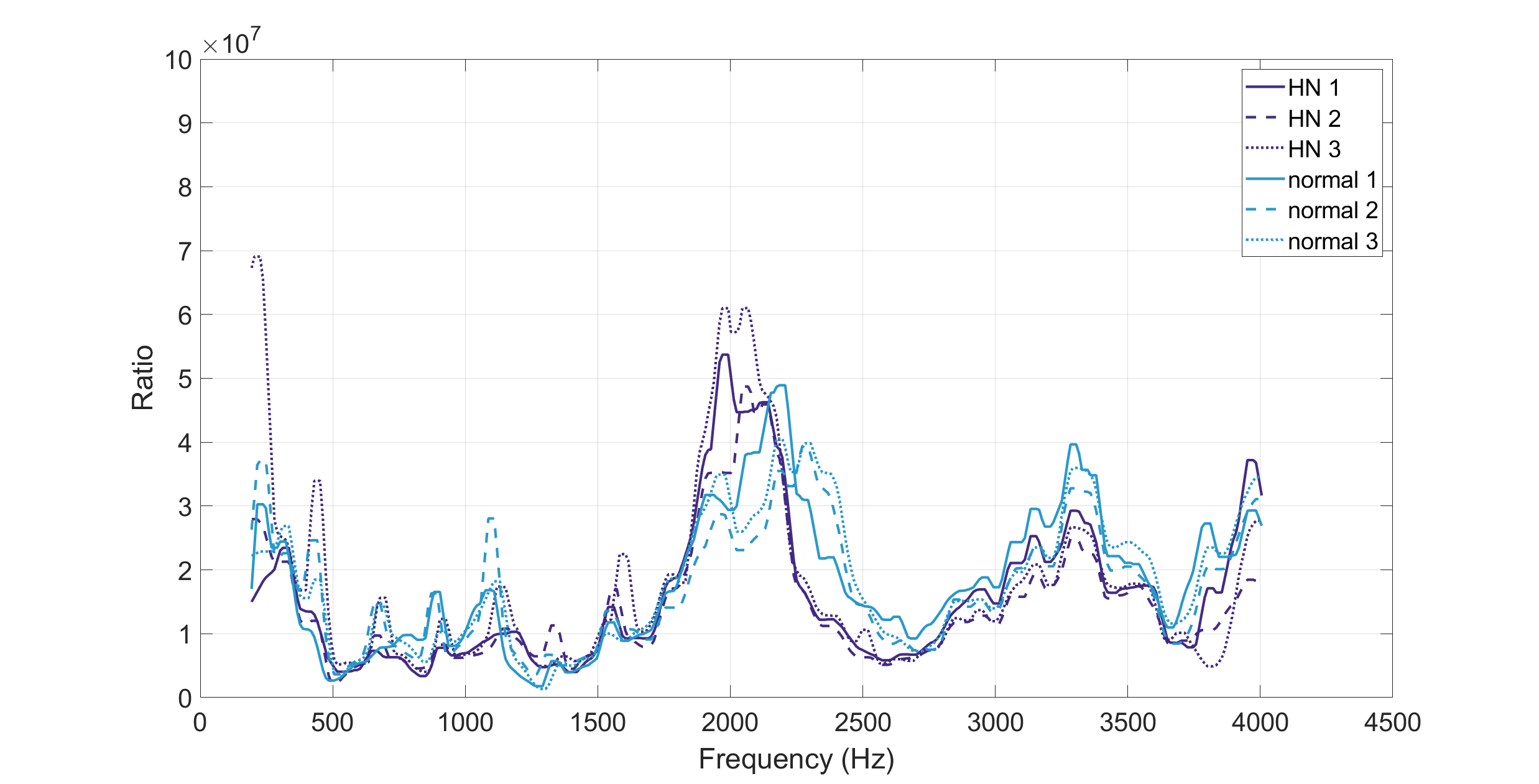 |
| --- |
| Figure 15: Transfer functions measured using the RMD at the nostrils for the vowel in ‘burn’. Colour denotes speech condition, line style denotes repetition. |

| 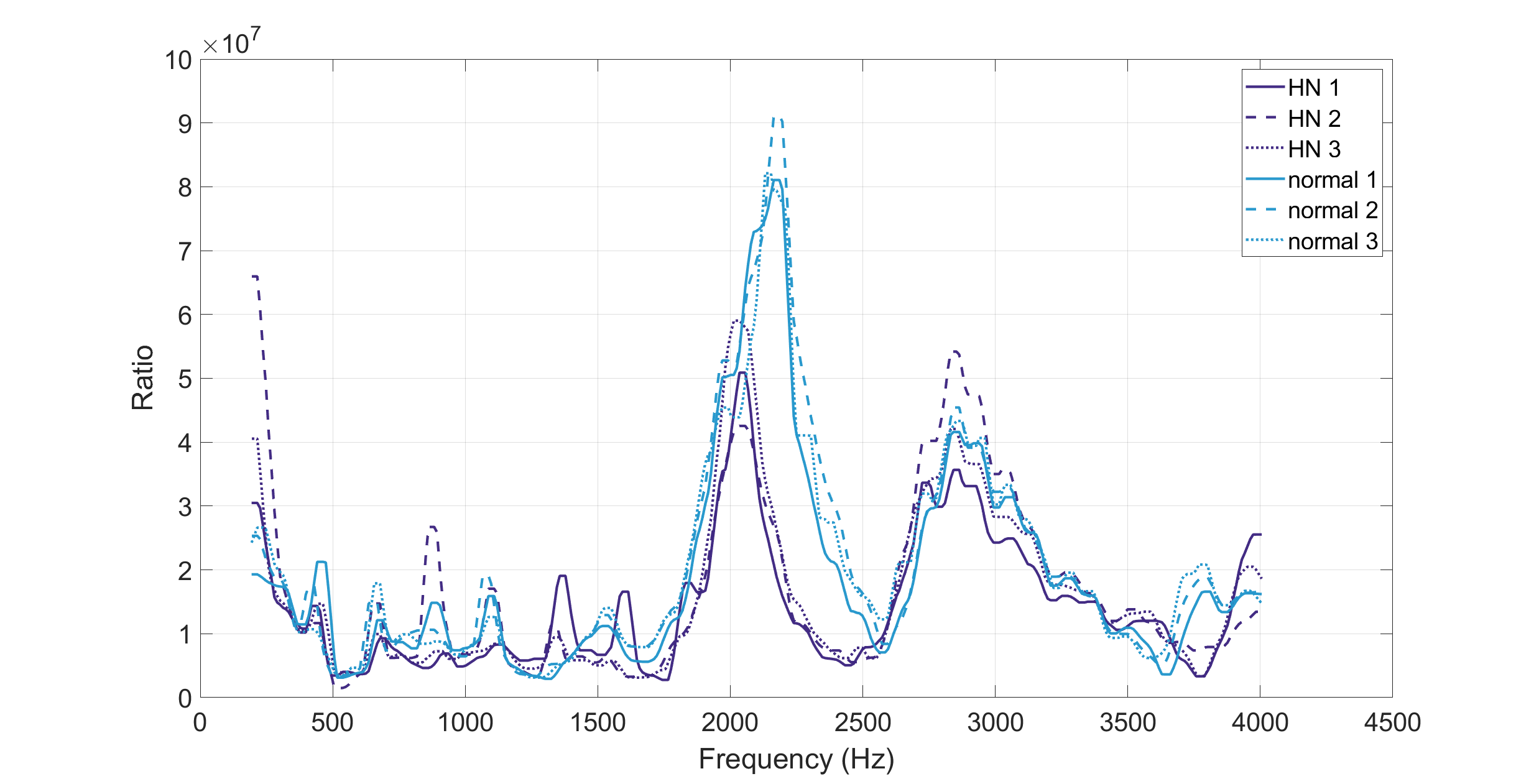 |
| --- |
| Figure 16: Transfer functions measured using the RMD at the nostrils for the vowel in ‘dawn’. Colour denotes speech condition, line style denotes repetition. |

| 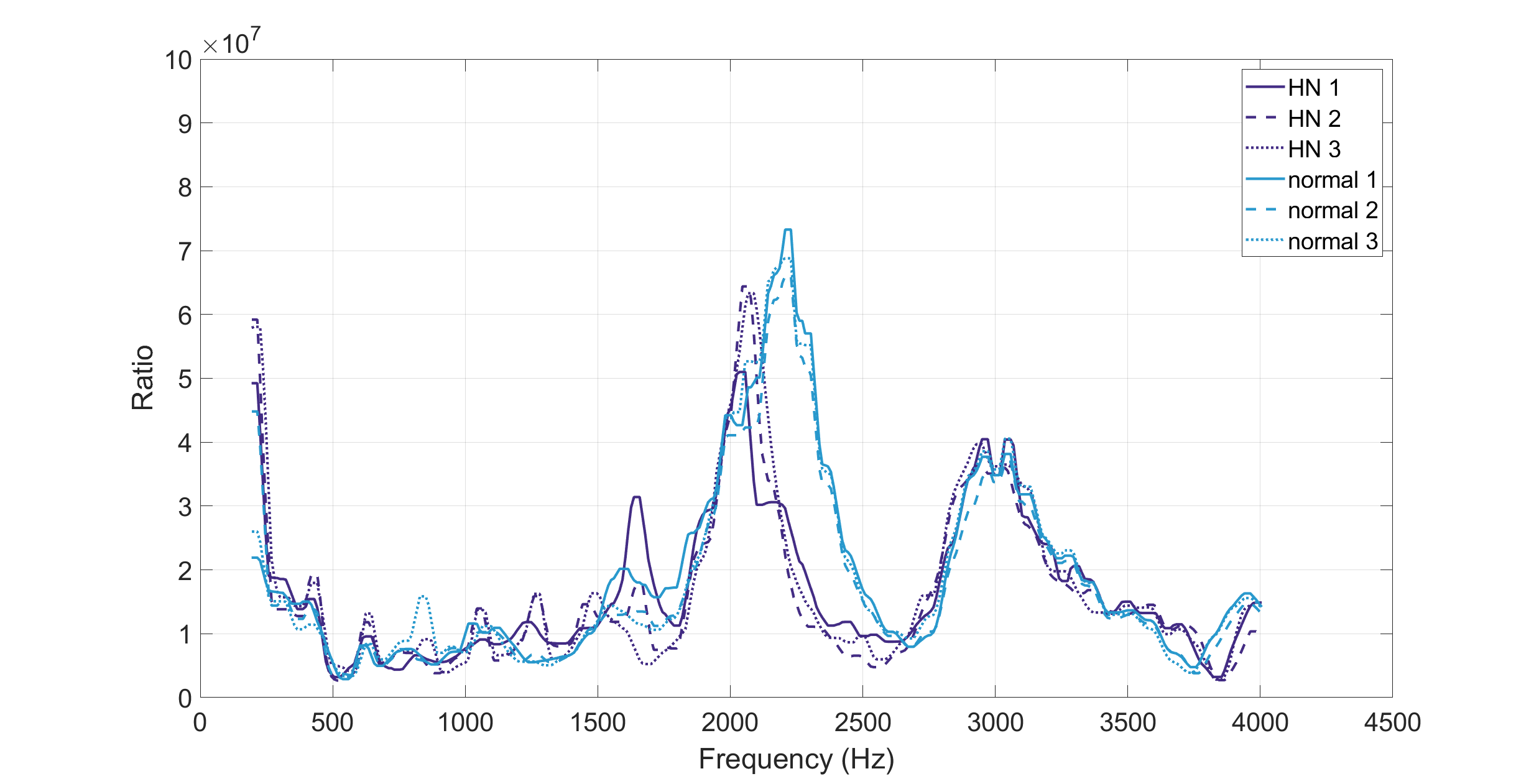 |
| --- |
| Figure 17: Transfer functions measured using the RMD at the nostrils for the vowel in ‘den’. Colour denotes speech condition, line style denotes repetition. |

| 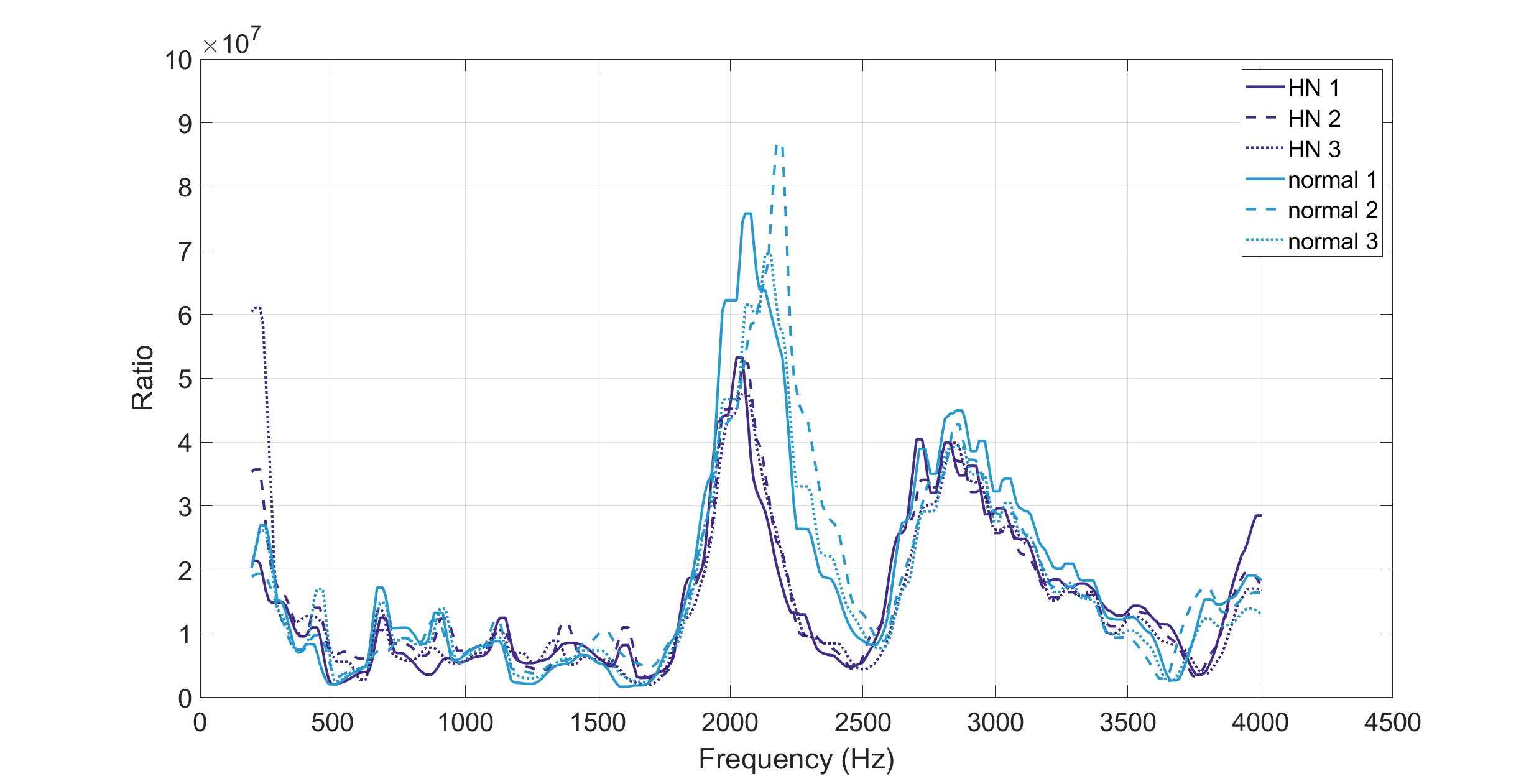 |
| --- |
| Figure 18: Transfer functions measured using the RMD at the nostrils for the vowel in ‘doll’. Colour denotes speech condition, line style denotes repetition. |

| 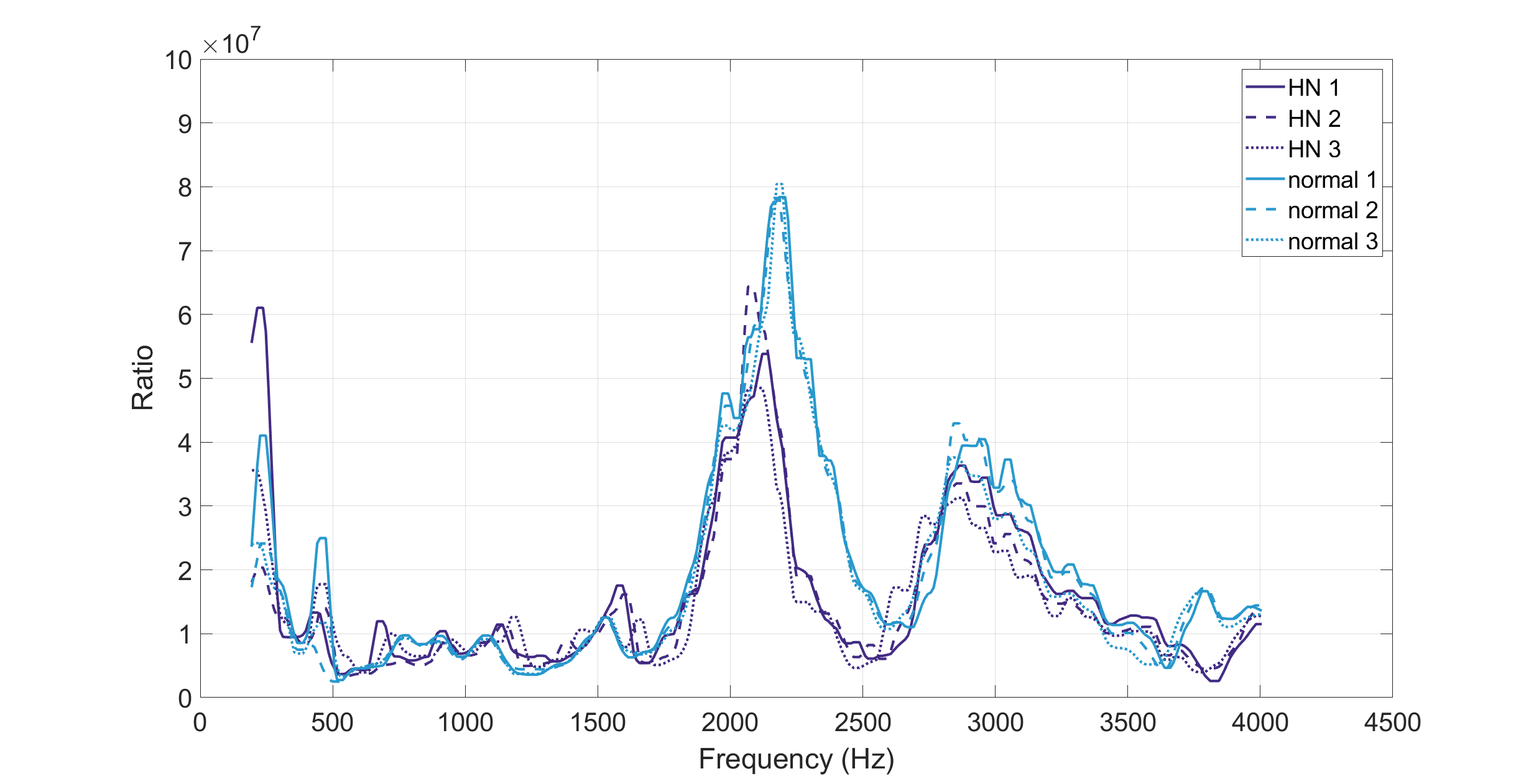 |
| --- |
| Figure 19: Transfer functions measured using the RMD at the nostrils for the vowel in ‘dune’. Colour denotes speech condition, line style denotes repetition. |
